# Work productivity by diseases diagnosed among workers: a study using large-scale claims data and survey data of workers in Japan

**DOI:** 10.1093/joccuh/uiaf055

**Published:** 2025-10-01

**Authors:** Takuya Maekawa, Kentaro Yamato, Norihiro Nakamichi, Yuka Kurita, Masami Nakai, Chihiro Osawa, Akiko Hatakama, Hitoshi Suzuki, Masaya Takahashi, Ryotaro Ishii, Takeo Nakayama

**Affiliations:** Medical Affairs, Otsuka Pharmaceutical Co, Ltd, Tokyo, Japan; Medical Affairs, Otsuka Pharmaceutical Co, Ltd, Tokyo, Japan; Department of Public Health, Graduate School of Medicine, Juntendo University, Tokyo, Japan; Medical Affairs, Otsuka Pharmaceutical Co, Ltd, Tokyo, Japan; Medical Affairs, Otsuka Pharmaceutical Co, Ltd, Tokyo, Japan; Medical Affairs, Otsuka Pharmaceutical Co, Ltd, Osaka, Japan; Medical Affairs, Otsuka Pharmaceutical Co, Ltd, Tokyo, Japan; Industry Management Division, DeSC Healthcare Inc, Tokyo, Japan; Industry Division, Data Horizon Co, Ltd, Hiroshima, Japan; Industry Management Division, DeSC Healthcare Inc, Tokyo, Japan; Industry Division, RWE Promotion Department, Data Horizon Co, Ltd, Hiroshima, Japan; Research Center for Overwork-Related Disorders, National Institute of Occupational Safety and Health, Kawasaki, Japan; Department of Neurology, Kyoto Prefectural University of Medicine, Kyoto, Japan; Department of Health Informatics, Graduate School of Medicine and School of Public Health, Kyoto University, Kyoto, Japan

**Keywords:** burden of disease, productivity, database, Japan, disease

## Abstract

**Objectives**: Japan faces the need for occupational health management based on an understanding of workers’ health and its impact on work productivity. Given a paucity of comprehensive studies, we conducted this study to investigate work productivity by diseases among workers of various occupations in Japan, by using a large-scale database.

**Methods**: This retrospective, descriptive study used pre-existing data derived from health insurance claims and 2 surveys conducted in 2021. The analysis included the data of ≥19-year-old current workers with response data to the questions regarding Work Productivity and Activity Impairment (WPAI). The WPAI of the target diseases, defined by claims diagnosis codes, was plotted against the 1-year prevalence of each disease. The cost of lost productivity was estimated based on the response data regarding the WPAI.

**Results**: Overall, 31 540 individuals participated, and the analysis showed that psychiatric disorders, headache, epilepsy, and insomnia had a high percentage of participants reporting any level of WPAI, although the prevalence of these diseases was low. We also explored the cost of lost productivity to supplement the interpretation of the overall impact of health problems; however, no clear trend was observed.

**Conclusions**: Many Japanese workers with psychiatric disorders, headaches, epilepsy, and insomnia have impaired work productivity and daily activities.

## Teaser

Key points

**What is already known on this topic:** Recent studies have reported that mental and behavioral disorders reduce work productivity; these studies, however, focused on specific populations and diseases, and examined only certain aspects of work productivity, requiring more comprehensive studies.

**What this study adds:** The present study builds upon previous research and provides an overview to enhance the understanding of work productivity among workers in Japan by including a large number of workers from diverse industries. We examined work productivity from multiple perspectives, in relation to a comprehensive range of diseases.

**How this study might affect research, practice, or policy:** The results showed that many workers with psychiatric disorders, headaches, epilepsy, and insomnia experience impaired work productivity and daily activities, underlining the importance of focused care for employees with such diseases.

## 1. Introduction

In Japan, the aging population and declining birthrate have expanded medical expenditure and shrunk the working population at an alarming rate, adding pressure on each worker’s health and work productivity for the sustainable development of enterprises and society.^1^ Employees’ physical and mental health conditions are closely related to their work productivity. The health problems of employees may manifest in the forms of absence from work (absenteeism) and reduced work performance even when present (presenteeism). Improving work productivity requires a coherent approach to occupational health management involving all stakeholders, including employers, insurers, health care providers, and government agencies. A comprehensive understanding of worker health and its impact on work productivity is a cornerstone for establishing an effective strategy.

Recent large-scale studies have provided valuable data on the health and work productivity of Japanese workers. Nagata et al^2^ reported that among approximately 12 000 employees of 4 pharmaceutical companies, the annual cost per person due to absenteeism was 520 US dollars (USD) and due to presenteeism was 3055 USD. They also demonstrated that of 34 common chronic diseases and conditions, mental and behavioral disorders, and musculoskeletal diseases incurred the highest total cost, mainly driven by presenteeism.^2^ In another study of approximately 80 000 employees from 10 companies by Inoue et al,^3^ disease burden, quantified in terms of mortality, long-term sickness absence, and retirement for illness, was highest for mental and behavioral disorders, followed by neoplasms and cardiovascular diseases.

A limitation of these studies, however, was that the target population and diseases were restricted,^2^^,^^3^ and the diseases attributed to presenteeism were defined based on self-reports and, thus, potentially included incorrect information.^2^ Moreover, in the report by Inoue et al,^3^ presenteeism was overlooked, although it is the largest contributor to the total monetary burden of employees’ ill health.^2^ To complement previous findings and obtain an overview of work productivity among Japanese workers, more comprehensive studies are needed. These studies should cover various health problems based on objective data and the resulting impact on the work faced by workers across many occupations from both subjective and objective perspectives.

Therefore, we conducted this study to investigate work productivity by diseases among workers of various occupations in Japan, using a large-scale database that holds claims data and survey data. We also aimed to quantify the cost of lost productivity by sex, age, and job category to supplement the interpretation of the impact of health problems in the workplace. Using this unique database facilitated the elicitation of credible information for disease diagnosis based on claims data while offering insight into workers’ perspectives of their work productivity based on survey data.

## 2. Methods

### 2.1. Study design and data source

This was a retrospective, descriptive study that used pre-existing anonymously processed data provided by DeSC Healthcare, Inc, Tokyo, Japan (DeSC). The data used in this study were derived from health insurance claims and health surveys of individuals insured by society-managed employment-based health insurance associations. These associations insure employees of large enterprises and their dependents. Under a contract with multiple health insurers in Japan, DeSC assembles data from the insurers to establish a large-scale database. DeSC also manages a mobile health application that supports health promotion for individuals insured by contracted insurance associations. DeSC conducts health surveys via Kencom® biannually in June and December, independently of this study. The survey data collected by each health insurer were provided to DeSC and integrated into a database. The claims data and survey data were linked and anonymously processed before the data were provided to the investigators.

Data on job categories, residential areas, and Work Productivity and Activity Impairment (WPAI) were extracted from data of the surveys conducted in June and December 2021 (referred to as the first and second surveys, respectively). The participants’ demographic characteristics (age and sex) at the time of survey and records of disease diagnosis were extracted from the claims data, along with insurance enrollment records, between January 1, 2021 and December 31, 2021.

### 2.2. Ethics statement

This retrospective study used secondary data that had been anonymized before data were provided to the investigators; thus, consent was not required for this study. This study was approved by the independent Ethics Committee of Otsuka Pharmaceutical Co, Ltd (approval no. 231004).

### 2.3. Study population

The target population was working individuals, identified by the following inclusion criteria: individuals who were insured by society-managed employment-based health insurance associations that consented to the secondary use of their medical data; were registered to Kencom®; responded to the first and/or second survey; and had the following data at the time of survey response: aged ≥19 years (based on insurance enrollment records), survey response that they were currently working, and responses to all the questions regarding WPAI (details in the section below). Participants were excluded if they answered in the first and/or second surveys that they were students or not currently working. Only the first survey data were used for participants, with both the first and second survey response data. Individuals aged ≥75 years were not included in the data because they were insured by different medical care systems for the advanced elderly in Japan.

### 2.4. Target disease

Two different definitions were adopted for the target diseases identified based on the International Classification of Diseases, 10th Revision (ICD-10) codes listed in Table S1.

Definition 1: Disease classification (ie, ICD-10 chapter title).

 

Definition 2: Diseases and conditions of interest were selected to reflect those ranked high in terms of years lived with a disability according to the Global Burden of Diseases, Injuries, and Risk Factors Study of 2016.^4^ We intended to include the following to be consistent with national surveys and health initiatives in Japan: (1) diseases designated as related to health and productivity management in a governmental promotional campaign for healthy working environments^1^; (2) diseases designated as characteristic of or frequently experienced by working women in a government-led national survey in Japan^5^; and (3) the 5 most common cancers among males and females in Japan.^6^ The disease selection was also informed by previous literature^7‑28^ and the clinical discretion of the investigators.

### 2.5. WPAI

To evaluate work productivity, the data from survey responses were used in the WPAI questionnaire-General Health (WPAI-GH).^29^ The WPAI-GH comprises 6 questions and measures WPAI due to “health problems” within the last 7 days, without addressing specific diseases or conditions. Based on the responses, WPAI was quantified in terms of 4 domain scores, expressed as an impairment percentage: (1) absenteeism: missed work hours due to health problems divided by the sum of hours missed and hours worked; (2) presenteeism: hours worked with reduced productivity due to health problems; (3) total work productivity impairment: the sum of absenteeism and presenteeism, that is, total hours missed and worked with reduced productivity; and (4) total activity impairment: interference in daily activities due to health problems. A higher percentage indicates a greater impairment.

### 2.6. Cost of lost work productivity

The lost work productivity was monetized as monthly absenteeism and presenteeism costs per person, based on the data of responses to the WPAI-GH. Monthly absenteeism cost was calculated using the following formula: absenteeism score (percentage) × average monthly salary by sex and age.^30^ Monthly presenteeism cost was calculated using the following formula: presenteeism score (percentage) × average monthly salary by sex and age.^30^

**Figure 1:**
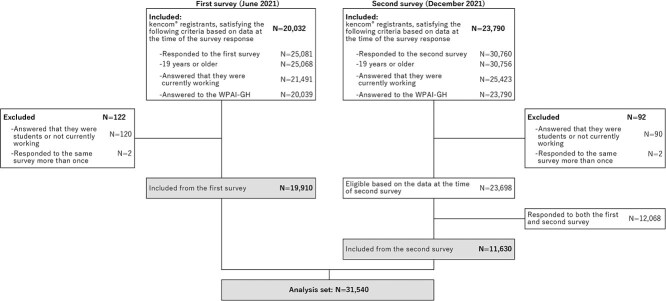
Participant disposition. WPAI-GH, Work Productivity and Activity Impairment questionnaire—General Health.

### 2.7. Statistical analysis

As indicated by the inclusion criteria, the analysis included participants whose response data for all questions on the WPAI-GH were available. The demographic characteristics of all participants included in the analysis are summarized descriptively. Categorical data are summarized as numbers and percentages, and continuous data as mean and SD. The trend of WPAI in the subgroups of target diseases (definitions 1 and 2) was visualized using a scatter plot, where the mean WPAI-GH score was plotted against the period prevalence of each disease. The period prevalence was calculated as the number of participants with a record of a specific disease more than once between January and December 2021 divided by the number of analysis sets. Scatter plots were generated for each sex. For disease definition 2, diagrams were created for age subgroups (≤29, 30-49, and ≥50 years). This analysis did not consider multimorbidity. Participants with more than 2 different diagnostic codes were counted for each disease, and the same WPAI-GH score was used for identified diseases. Diseases recorded in ˂10 participants are not plotted in the diagrams. The cost of lost work productivity was calculated and summarized according to sex, age, and job category. As the analysis included participants whose response data to the WPAI-GH were available, missing data were not imputed. All analyses were performed using SAS version 9.4, Release 7.0 (SAS Institute Inc, Cary, NC, USA).

## 3. Results

### 3.1. Participant disposition and characteristics

Figure 1 shows the participants’ dispositions. Among the 25 081 participants in the first survey, 19 910 met the inclusion and/or exclusion criteria and were included in the analysis. Among the 30 760 participants in the second survey, 11 630 who met the criteria and responded only to the second survey were included, yielding a total of 31 540 participants.

Table 1 summarizes the demographic characteristics of the participants. Males were predominant (72.4%). Most participants were aged ≥50 years (50.8%), and only a fraction was aged ≤29 years (5.9%); the mean (SD) age of the analysis set was 48.1 (10.1) years old. The most common job category was clerical workers (25.3%), followed by professional/technical workers (24.8%) and managerial positions (17.9%).

**Table 1:** Baseline characteristics.^a^

**Characteristics**	**Total (*n* = 31 540)**
Sex^b^	
Male	22825 (72.4)
Female	8715 (27.6)
Age,^b^ y	
Mean (SD)	48.1 (10.1)
≤29	1851 (5.9)
30-49	13681 (43.4)
≥50	16008 (50.8)
Job category^c^	
Professional/technical workers	7816 (24.8)
Managerial positions	5657 (17.9)
Clerical workers	7988 (25.3)
Marketing workers	2838 (9.0)
Sales workers	239 (0.8)
Transportation and communication workers	66 (0.2)
Security workers	96 (0.3)
Skilled trade	1975 (6.3)
Agriculture, forestry, and fishery	45 (0.1)
Service (qualification required)	228 (0.7)
Service (qualification not required)	1011 (3.2)
Others	3581 (11.4)
Prefecture of residence^c^^,^^d^	
Kanagawa	5680 (18.0)
Tokyo	4046 (12.8)
Hyogo	3266 (10.4)
Osaka	1921 (6.1)
Saitama	1892 (6.0)

a Data are presented as frequencies and percentages unless otherwise indicated. Percentages may not sum to 100% because of rounding.

b Defined based on the claims data, including insurance enrollment records.

c Defined based on the survey response data.

d Top 5 areas are listed.

### 3.2. WPAI by disease classifications (definition 1)

Initially, we planned to assess the mean WPAI-GH score; however, the data revealed that many participants scored 0. Therefore, the score was dichotomized to 0% or >0%, and the percentage of participants with a WPAI-GH score of >0% was plotted. As a reference, the percentage of participants with a WPAI-GH score >0% among participants without a record of health insurance claims issuance (ie, those who had no diseases) between January and December 2021 was also plotted.

**Figure 2:**
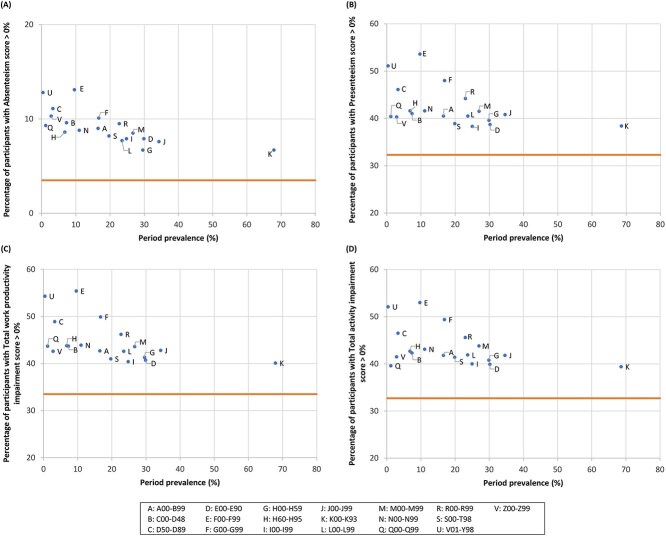
Percentage of participants with a WPAI-GH score of >0% in terms of absenteeism (A), presenteeism (B), total work productivity impairment (C), and total activity impairment (D) by the period prevalence of diseases (definition 1: Disease classification) among male workers. The period prevalence was calculated as the proportion of participants with a record of a specific disease more than once between January and December 2021. The horizontal line indicates the WPAI-GH score of the reference group, defined as participants without a record of health insurance claim issuance (ie, those who had no diseases) between January and December 2021. Diseases recorded in <10 participants are not plotted in the diagrams. ICD-10 codes and the corresponding disease category are as follows: A00-B99: Certain infectious and parasitic diseases; C00-D48: Neoplasms; D50-D89: Diseases of the blood and blood-forming organs and certain disorders involving the immune mechanism; E00-E90: Endocrine, nutritional, and metabolic diseases; F00-F99: Mental and behavioral disorders; G00-G99: Diseases of the nervous system; H00-H59: Diseases of the eye and adnexa; H60-H95: Diseases of the ear and mastoid process; I00-I99: Diseases of the circulatory system; J00-J99: Diseases of the respiratory system; K00-K93: Diseases of the digestive system; L00-L99: Diseases of the skin and subcutaneous tissue; M00-M99: Diseases of the musculoskeletal system and connective tissue; N00-N99: Diseases of the genitourinary system; Q00-Q99: Congenital malformations, deformations, and chromosomal abnormalities; R00-R99: Symptoms, signs and abnormal clinical and laboratory findings, not elsewhere classified; S00-T98: Injury, poisoning, and certain other consequences of external causes; V01-Y98: External causes of morbidity and mortality; Z00-Z99: Factors influencing health status and contact with health services. ICD-10, International Classification of Diseases, 10th Revision; WPAI-GH, Work Productivity and Activity Impairment questionnaire—General Health.

**Figure 3:**
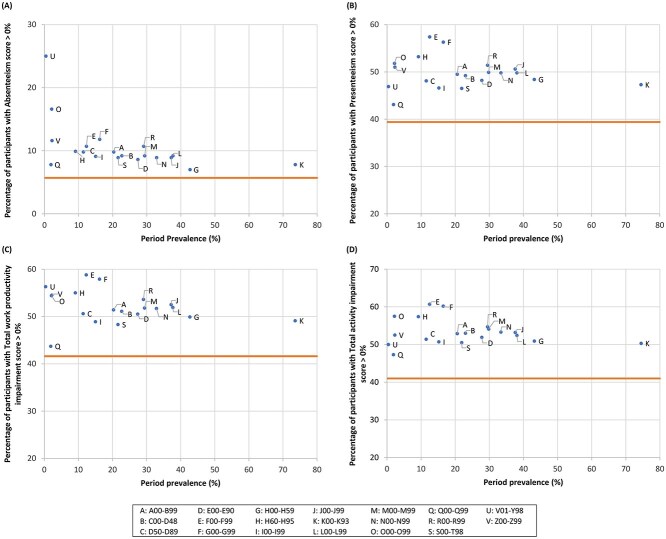
Percentage of participants with a WPAI-GH score of >0% in terms of absenteeism (A), presenteeism (B), total work productivity impairment (C), and total activity impairment (D) by the period prevalence of diseases (definition 1: Disease classification) among female workers. The period prevalence was calculated as the proportion of participants with a record of a specific disease more than once between January and December 2021. The horizontal line indicates the WPAI-GH score of the reference group, defined as participants without a record of health insurance claim issuance (ie, those who had no diseases) between January and December 2021. Diseases recorded in <10 participants are not plotted in the diagrams. ICD-10 codes and the corresponding disease category are as follows: A00-B99: Certain infectious and parasitic diseases; C00-D48: Neoplasms; D50-D89: Diseases of the blood and blood-forming organs and certain disorders involving the immune mechanism; E00-E90: Endocrine, nutritional, and metabolic diseases; F00-F99: Mental and behavioral disorders; G00-G99: Diseases of the nervous system; H00-H59: Diseases of the eye and adnexa; H60-H95: Diseases of the ear and mastoid process; I00-I99: Diseases of the circulatory system; J00-J99: Diseases of the respiratory system; K00-K93: Diseases of the digestive system; L00-L99: Diseases of the skin and subcutaneous tissue; M00-M99: Diseases of the musculoskeletal system and connective tissue; N00-N99: Diseases of the genitourinary system; O00-O99: Obstetric and gynecological diseases; Q00-Q99: Congenital malformations, deformations, and chromosomal abnormalities; R00-R99: Symptoms, signs, and abnormal clinical and laboratory findings, not elsewhere classified; S00-T98: Injury, poisoning, and certain other consequences of external causes; V01-Y98: External causes of morbidity and mortality; Z00-Z99: Factors influencing health status and contact with health services. ICD-10, International Classification of Diseases, 10th Revision; WPAI-GH, Work Productivity and Activity Impairment questionnaire—General Health.

Figures 2 and 3 illustrate the percentage of participants with a WPAI-GH score of >0% according to the prevalence of disease classification (definition 1) among males and females, respectively. The actual values are presented in Tables S2 and S3, respectively. Overall, the percentage of participants with a WPAI-GH score >0% was higher in all the disease categories than in the reference group (no diseases). Among male participants, mental and behavioral disorders (F00-F99) as well as external causes of morbidity and mortality (V01-Y98) had the highest percentage of participants with WPAI-GH scores >0% across the domains, whereas the period prevalence of these diseases was low. In females, high-ranking diseases were generally similar between absenteeism and total work productivity impairment regarding the distribution where external causes of morbidity and mortality (V01-Y98), and obstetric and gynecological diseases (O00-O99) were positioned high. The pattern was also similar between presenteeism and total activity impairment; mental and behavioral disorders (F00-F99), diseases of the nervous system (G00-G99), obstetric and gynecological diseases (O00-O99), and diseases of the ear and mastoid process (H60-H95) ranked high.

### 3.3. ***WPAI by diseases of interest (*** ***definition 2*** ***)***

**Figure 4:**
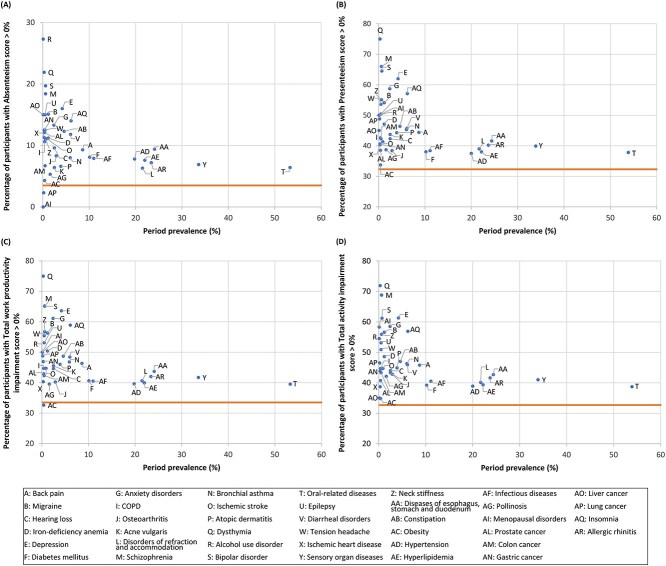
Percentage of participants with a WPAI-GH score of >0% in terms of absenteeism (A), presenteeism (B), total work productivity impairment (C), and total activity impairment (D) by the period prevalence of diseases (definition 2: Diseases and conditions of interest) among male workers. The period prevalence was calculated as the proportion of participants with a record of a specific disease more than once between January and December 2021. The horizontal line indicates the WPAI-GH score of the reference group, defined as participants without a record of health insurance claim issuance (ie, those who had no diseases) between January and December 2021. Diseases recorded in <10 participants are not plotted in the diagrams. WPAI-GH, Work Productivity and Activity Impairment questionnaire—General Health.

**Figure 5:**
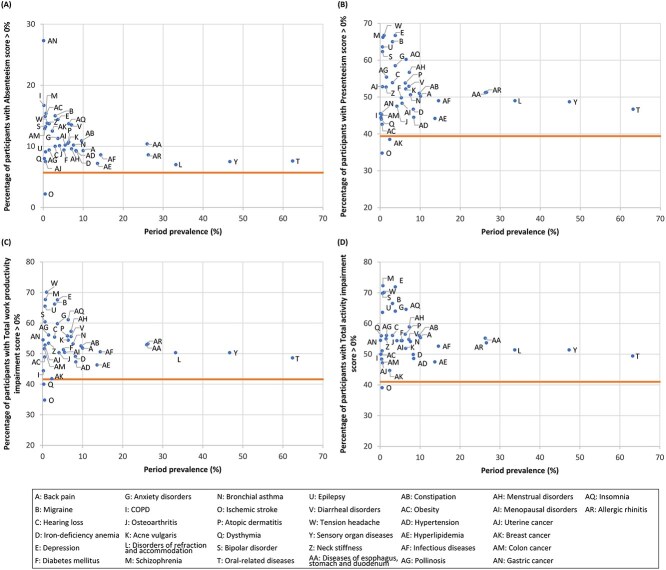
Percentage of participants with a WPAI-GH score of >0% in terms of absenteeism (A), presenteeism (B), total work productivity impairment (C), and total activity impairment (D) by the period prevalence of diseases (definition 2: Diseases and conditions of interest) among female workers. The period prevalence was calculated as the proportion of participants with a record of a specific disease more than once between January and December 2021. The horizontal line indicates the WPAI-GH score of the reference group, defined as participants without a record of health insurance claim issuance (ie, those who had no diseases) between January and December 2021. Diseases recorded in <10 participants are not plotted in the diagrams. WPAI-GH, Work Productivity and Activity Impairment questionnaire—General Health.

Among the diseases of interest (definition 2), common diseases, such as oral-related diseases, sensory organ diseases, and refraction and accommodation disorders, had a low percentage of participants with WPAI-GH scores >0% in both males (Figure 4) and females (Figure 5). The actual values are presented in Tables S4 and S5, respectively. Although disease prevalence was low, psychiatric disorders (including schizophrenia, bipolar disorder, and depression), migraines, tension headache, neck stiffness, epilepsy, and insomnia ranked highly, generally across the domains in both sexes (Figures 4 and 5). Uniquely for women, menstrual disorders ranked relatively high (Figure 5). The trend of high-ranking diseases was relatively similar across the age subgroups of each sex (Figures S1-S6). Only for the absenteeism domain among females were gastric, breast, and colon cancers ranked highly, but their prevalence was low (Figure 5).

### 3.4. Cost of lost work productivity

The median cost of lost work productivity per person, in terms of both absenteeism and presenteeism costs, was 0 yen in almost all subgroups; the cost of third quartile was also 0 yen for absenteeism. The mean (SD) monthly absenteeism cost per person was 5569.3 (31 405.2) yen among males, ranging from 3587.3 (18 164.2) to 5699.0 (32 609.1) yen across the age subgroups; and 4403.8 (21 950.8) yen among females, ranging from 3357.6 (18 616.9) to 4719.9 (22 145.2) yen across the age subgroups (Table S6). The mean (SD) monthly presenteeism cost per person was 39 935.8 (72 594.4) yen among males, ranging from 35 387.9 (70 571.1) to 46 563.4 (76 401.4) yen across the age subgroups; and 39 004.5 (59 205.1) yen among females, ranging from 30 555.9 (52 191.4) to 46 589.4 (57 886.3) yen across the age subgroups (Table S7).

## 4. Discussion

This study aimed to illustrate a real-world overview of work productivity among Japanese workers using large-scale claims data and survey response data. Using this database enabled us to include individuals from various occupations with diverse diseases and conditions. Although the period prevalence was relatively low, a high percentage of males and females with psychiatric disorders, headaches, epilepsy, and insomnia had WPAI-GH scores of >0%. Oral diseases, sensory organ diseases, and refraction and accommodation disorders were commonly identified in men and women, and only a relatively small percentage had a WPAI-GH score of >0%.

Previous studies also identified psychiatric disorders, headaches, and insomnia as major drivers of work productivity decrement among Japanese workers.^2^^,^^31^ Other studies also illustrated the impact of these diseases; for instance, studies based on self-reported surveys for headaches consistently captured impaired work productivity and daily activities among patients with migraine^32^ and other headache types.^33^ Workers with diagnosed depression had greater work productivity impairment than those with undiagnosed depression.^34^ Menstrual problems, including dysmenorrhea, were reported to affect absenteeism and productivity at work.^35^ Methodological differences must be acknowledged when interpreting the present results in the context of previous studies. For example, previous studies^2^^,^^31‑35^ relied on self-reported data, which may capture a broader range of individuals, including those who were undiagnosed or untreated. In contrast, the present study used claims data, which more accurately reflect physician-diagnosed conditions but naturally exclude individuals who do not seek medical care. Furthermore, the dichotomization of the WPAI-GH score in the present study may not allow direct comparison with studies that assessed work productivity impairment by mean scores. Considering the small percentage of participants who scored >0% on the WPAI-GH, the present analysis population (ie, currently working and can respond to the survey) was expected to have mild conditions, if any, and be less likely to require long-term sick leave. However, as discussed above, high-ranking diseases in terms of the percentage of participants with WPAI-GH scores >0% generally corresponded with diseases associated with lost work productivity in previous studies, collectively underlining the importance of focused care for employees with such diseases.

Diseases that ranked high in the percentage of participants with WPAI-GH scores >0% were characterized by low physician consultation rates. Previous studies on headaches,^36^ menstrual problems,^35^ and mental disorders, including depression,^37^ have suggested that many patients do not seek medical help. They are underdiagnosed or undertreated despite symptom manifestations and impact on their lives. Various barriers hinder patients from seeking appropriate care from health care professionals, including a lack of recognition that their diseases and conditions require professional treatment,^36^^,^^38^ stigma,^39^ and, particularly for menstrual disorders, reluctance for consultation and examination.^38^ Using health insurance claims data to define diseases, we cannot infer how many participants did not consult physicians while having symptoms. Nevertheless, based on previous findings, attention should also be paid to employees who do not visit hospitals by raising awareness of diseases that are likely to impact their work performance and encouraging them to receive treatment from physicians.

A previous systematic review found that work performance was more impaired in cancer survivors, particularly for those diagnosed within 5 years and with complications related to cancer and treatment.^40^ However, the results of the present study did not demonstrate productivity impairment in many workers with cancer, relative to other diseases with high percentages, except for the absenteeism domain among females. The discrepancy with previous findings may partly be due to the target population of this study being active workers at survey and being more likely to have mild symptoms without pain or have their symptoms improved by treatment. These results may also be attributed to the small sample size of this disease group, which warrants further confirmation.

The cost estimation of lost work productivity indicated that the median monthly cost was zero for most subgroups; the SD exceeded the mean, reflecting a heavily skewed distribution toward zero (no impairment) for all subgroups, with a small subset of respondents reporting substantial impairment. An underlying factor for this tendency may be the inclusion of participants that were currently working, which likely excludes those on long-term sick leave and those with severe impairments in work productivity or daily functioning. Therefore, it is difficult to confirm the actual trend in the cost of lost work productivity of all workers based on the mean value, and further studies on the economic implications of WPAI are warranted. However, it may be inferred from the present results that, regardless of sex or age, the presenteeism cost was higher than the absenteeism cost, as was reported previously in Japan, with a similar absenteeism:presenteeism ratio.^2^

Attention should be paid to these limitations when interpreting the results of this study. First, considering that Kencom® users were included, the analysis population may be more health conscious, potentially maintaining higher occupational health and higher work productivity than nonusers. In addition, individuals who are ≥75 years old are not insured by society-managed employment-based health insurance associations, and thus were not included in this study. However, this study’s analysis population predominantly comprised males in their 40s and 50s, which reflects the actual situation of the working population in Japan. Second, a limitation inherent to the use of claims data is that the data were originally collected for reimbursement purposes. Diseases in claims data may not necessarily correspond to the actual clinical diagnosis and potentially include diagnoses recorded in the context of preventive screenings (eg, preventive care for periodontal disease recorded as an oral-related disease diagnosis in claims data). Claims data may not cover all the relevant information. For instance, pregnancy is assumed to be related to work performance for some women; however, check-ups for normal pregnancy and delivery are not covered by public health insurance, and relevant claims data are not available in the database. Moreover, as some diseases with a high burden are known to be underdiagnosed, the nature of claims data, which exclude individuals who do not seek medical care, may have led to the underestimation of such conditions. Third, WPAI was measured solely on a self-report scale, and hours missed and worked may be reported incorrectly, potentially over- or underestimating the impact on work productivity. Moreover, the results of this study could not identify which diseases reduced work productivity since the WPAI-GH questionnaire does not specify which diseases or conditions to assume when answering the questions, and our analysis did not consider multimorbidity. Finally, we dichotomized the scores into 0% and >0%, as many respondents scored 0% on the WPAI. Although this analysis enabled us to assess the presence of WPAI, it did not allow us to evaluate its magnitude, highlighting the need for further investigation.

## 5. Conclusion

Among workers in Japan, many with psychiatric disorders, headaches, epilepsy, and insomnia have impaired work productivity and daily activities. Based on health insurance claims data and survey response data of many workers from diverse job categories, the data presented may facilitate a preliminary understanding of actual work productivity of diverse diseases among Japanese workers.

## Supplementary Material

Web_Material_uiaf055

## Data Availability

The data underlying this article will be shared on reasonable request to the corresponding author.
