# Supplementary material for: Work productivity by diseases diagnosed among workers: a study using large-scale claims data and survey data of workers in Japan
Source: J Occup Health. 2025 Oct 1;67(1):uiaf055. doi: 10.1093/joccuh/uiaf055 (PMC12574326; doi:10.1093/joccuh/uiaf055)
Supplement: Web_Material_uiaf055 [file web_material_uiaf055.docx]

# **Supplementary Figure 1. Percentage of participants with a WPAI-GH of >0 in terms of absenteeism (A), presenteeism (B), total work productivity impairment (C), and total activity impairment (D) by the period prevalence of diseases (definition 2: diseases and conditions of interests) among male workers of ≤29 years old**


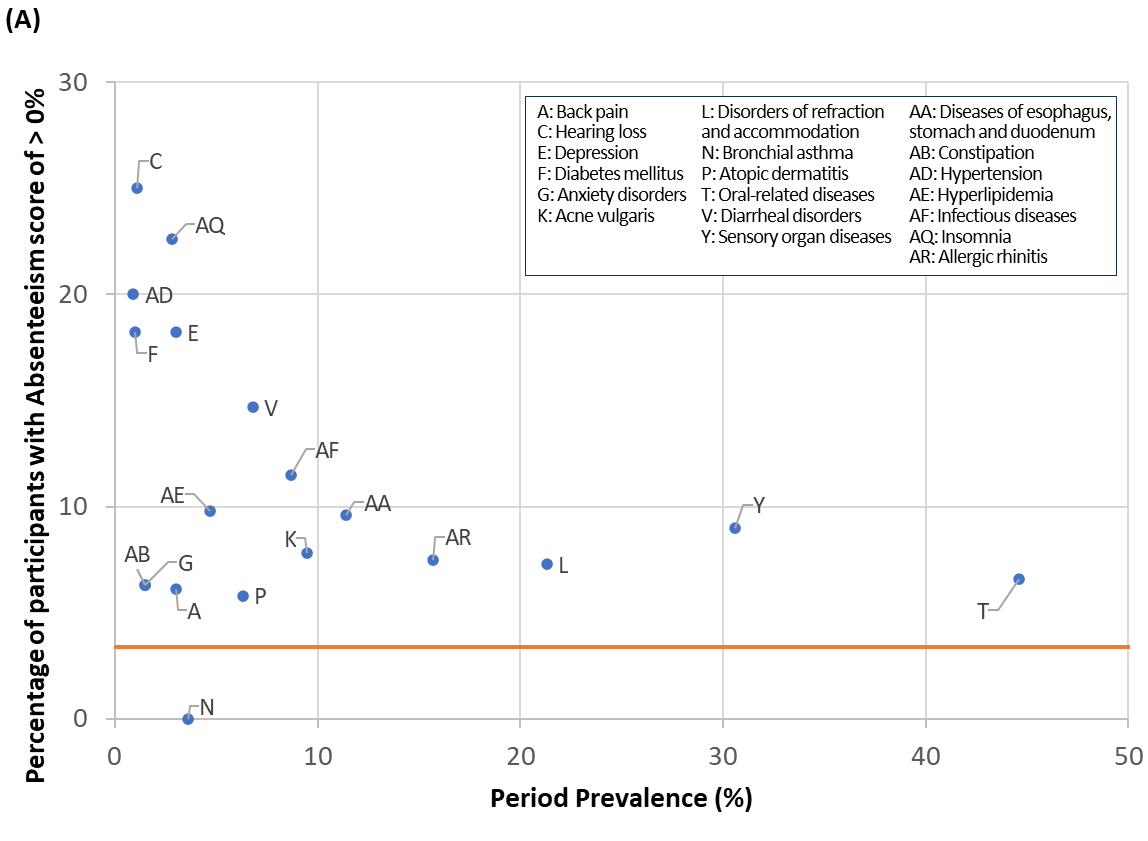


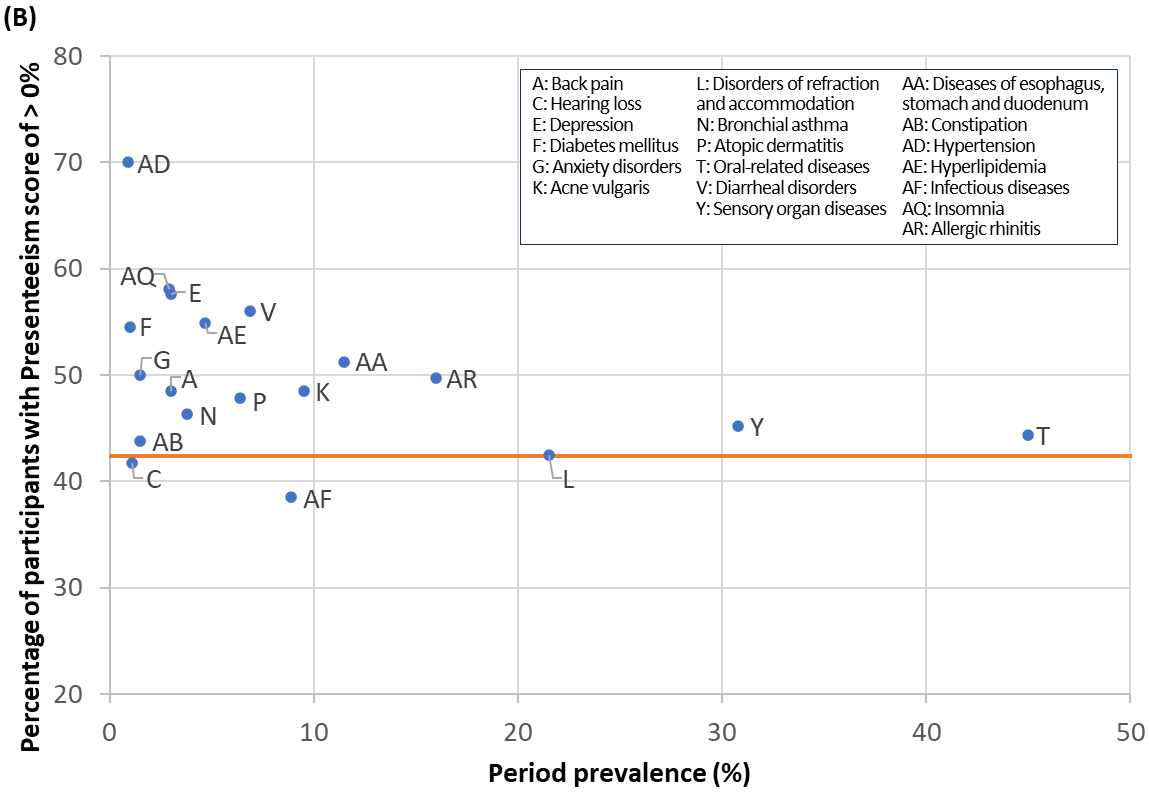


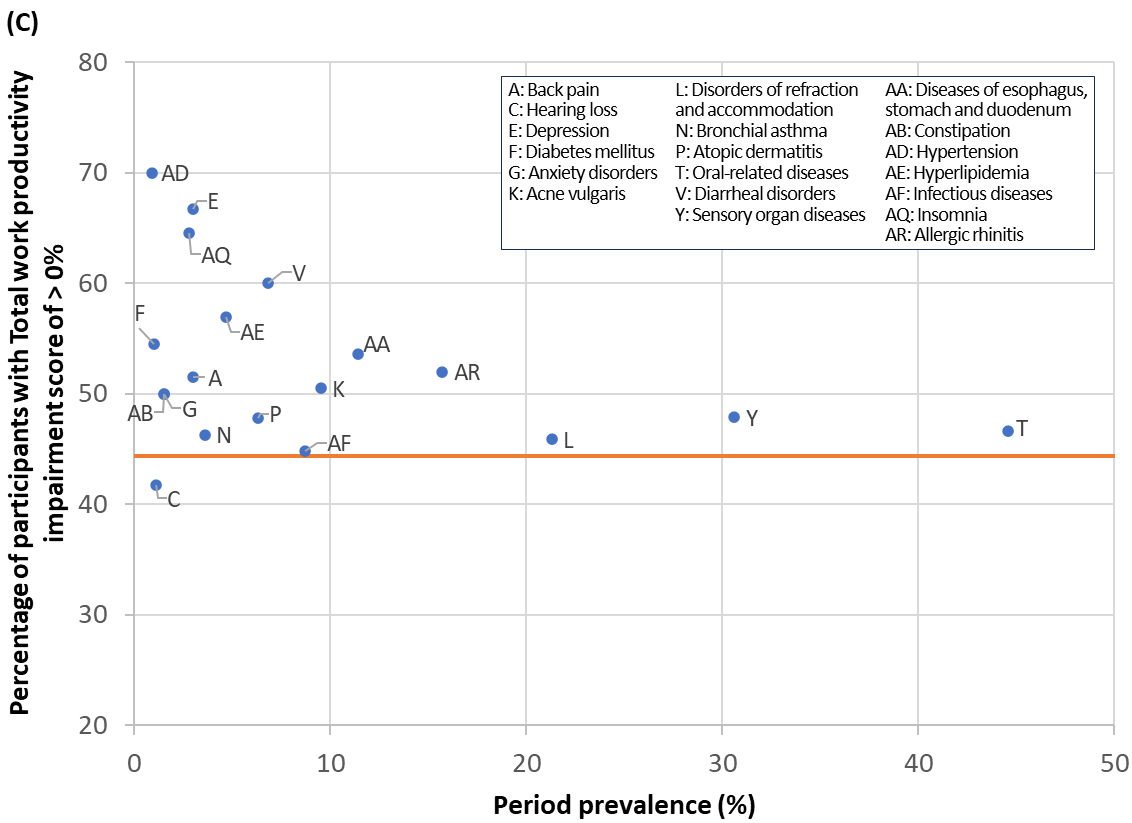


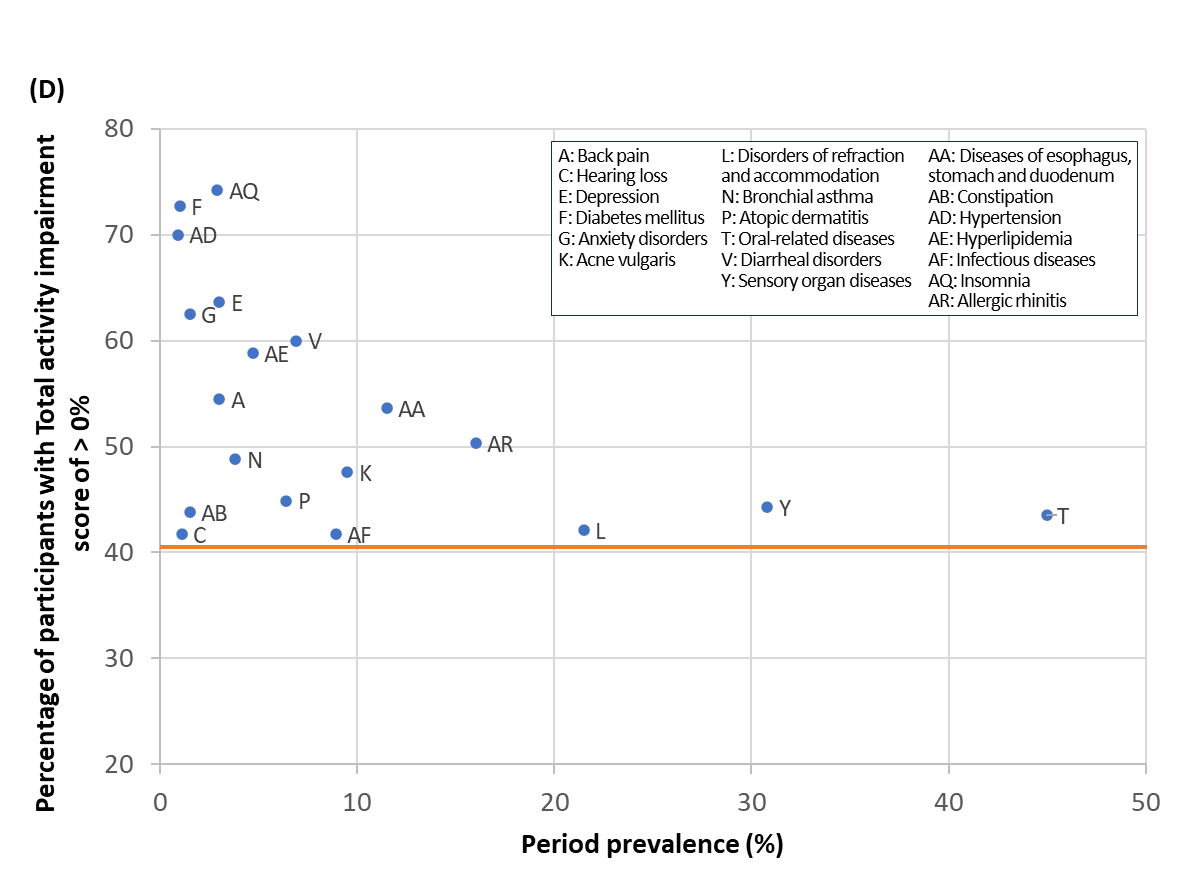


WPAI-GH, Work Productivity and Activity Impairment questionnaire-General Health

The period prevalence was calculated as the proportion of participants with a record of a specific disease more than once between January and December 2021.

The orange line indicates the WPAI-GH score of the reference group, defined as participants without a record of health insurance claim issuance (i.e., those who had no diseases) between January and December 2021.
Diseases recorded in <10 participants are not plotted in the diagrams.

# **Supplementary Figure 2. Percentage of participants with a WPAI-GH score of >0% in terms of absenteeism (A), presenteeism (B), total work productivity impairment (C), and total activity impairment (D) by the period prevalence of diseases (definition 2: diseases and conditions of interests) among male workers of 30–49 years old**


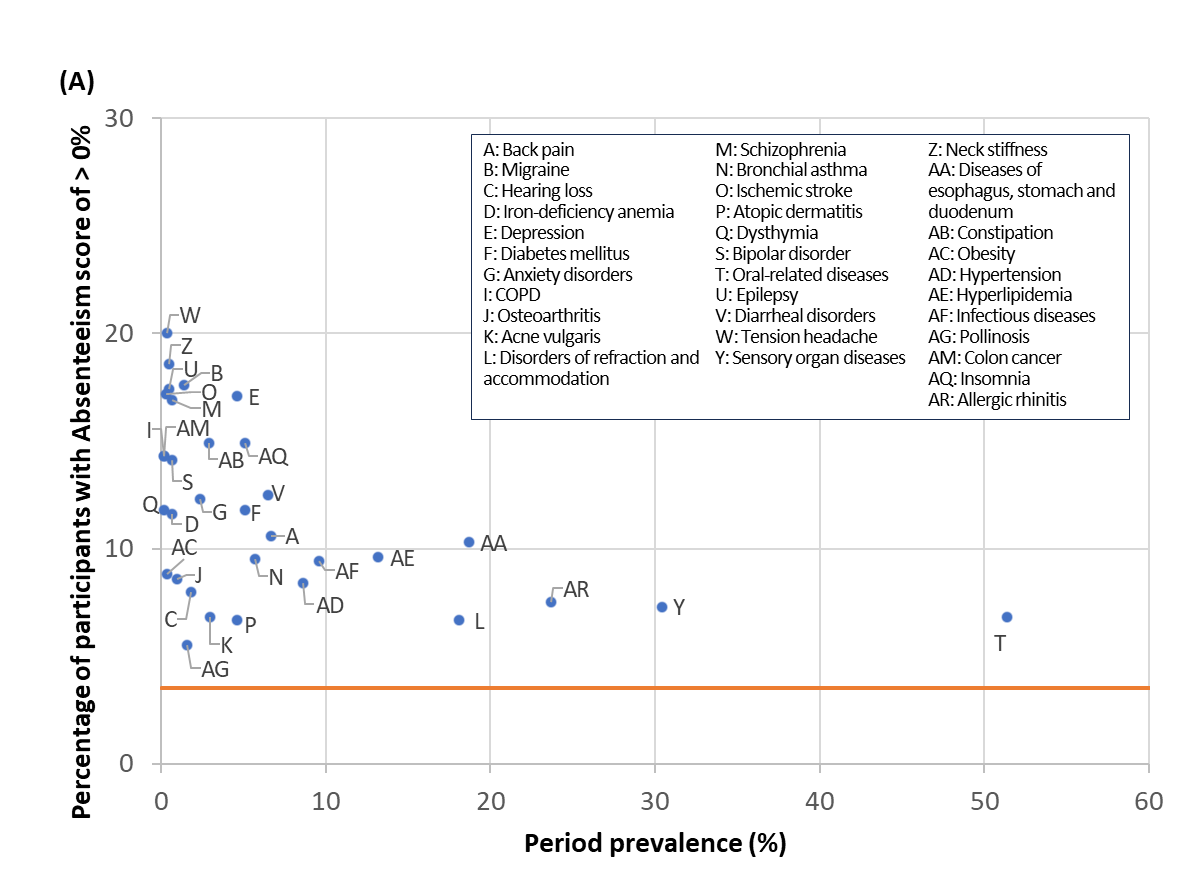


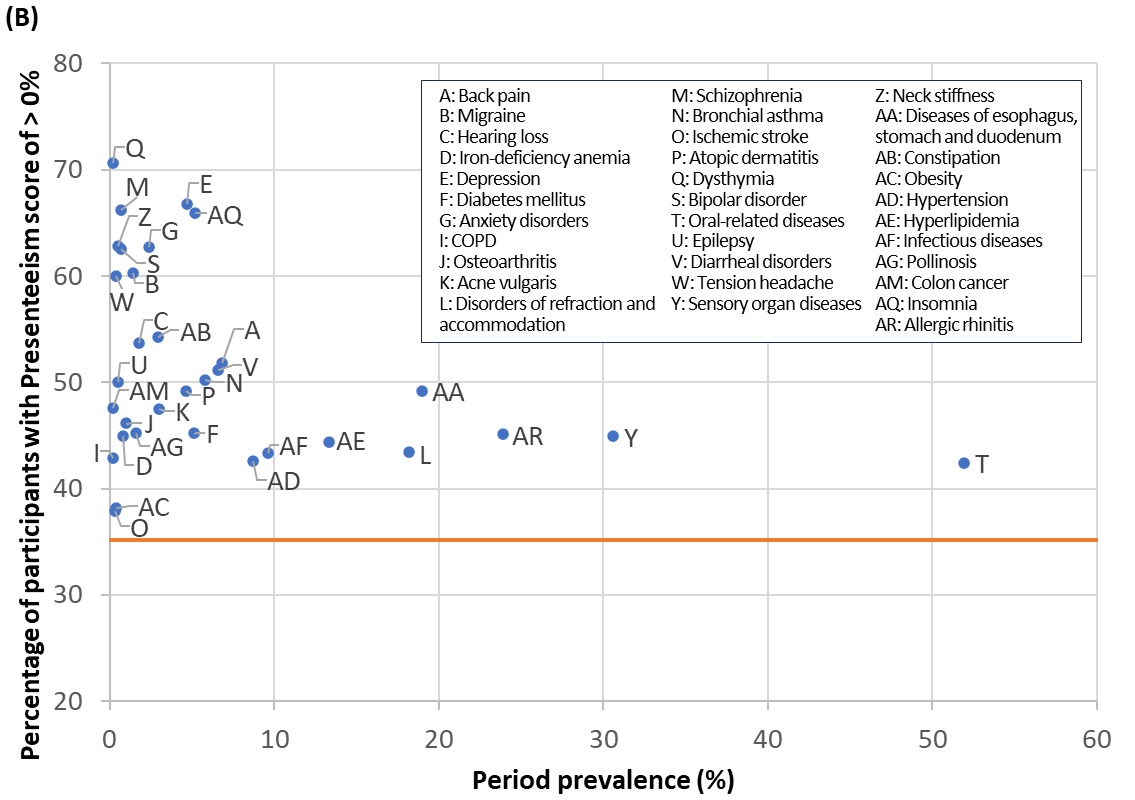


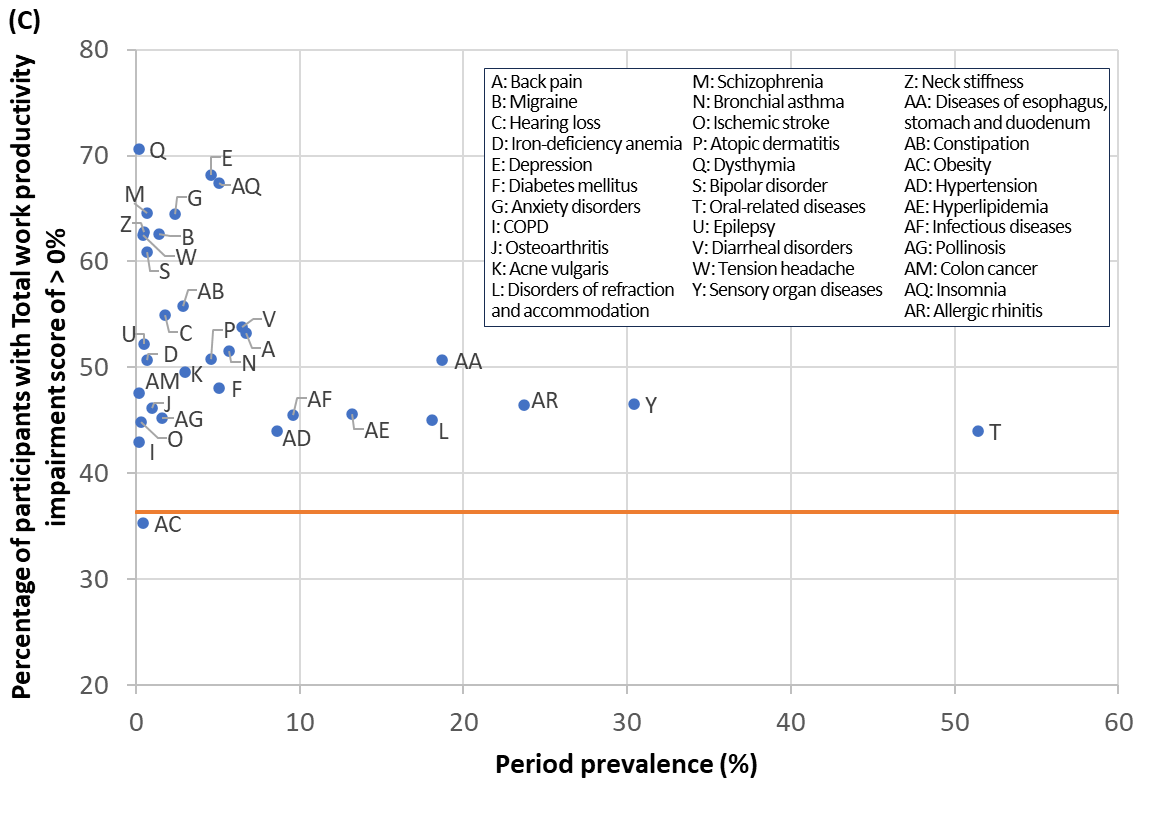

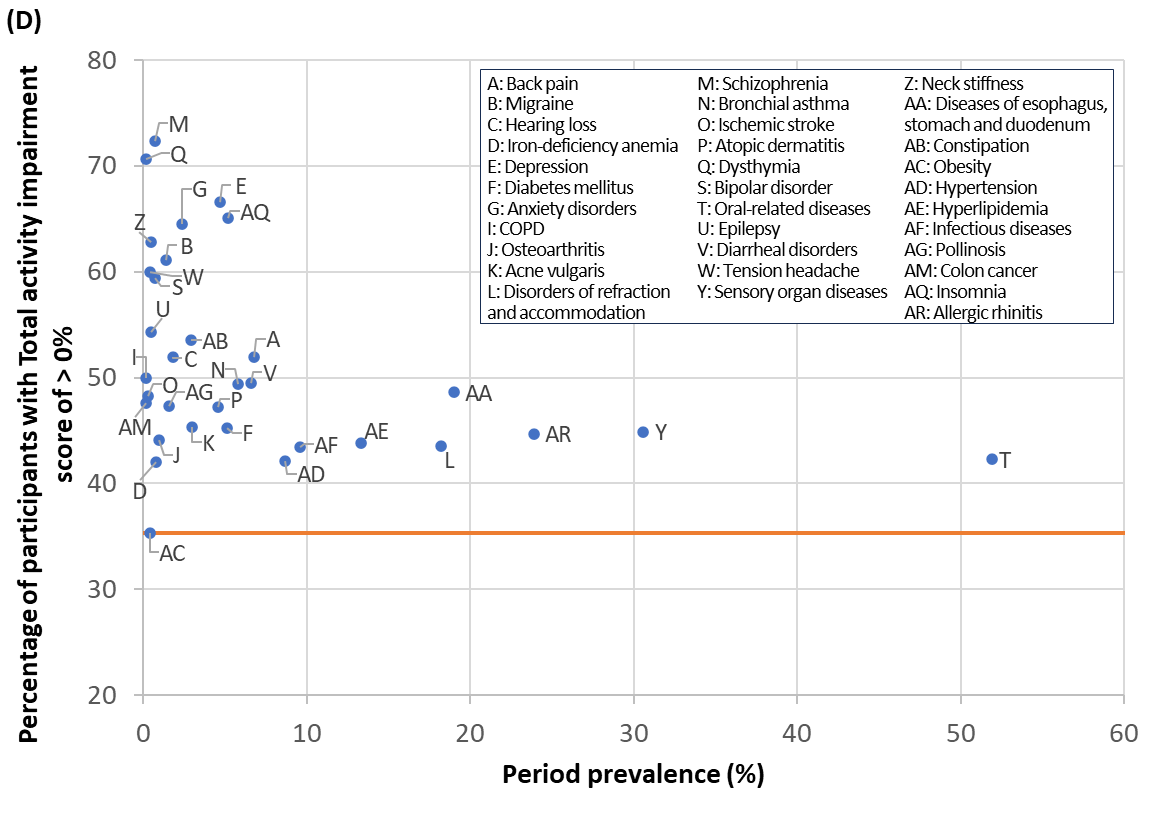


WPAI-GH, Work Productivity and Activity Impairment questionnaire-General Health

The period prevalence was calculated as the proportion of participants with a record of a specific disease more than once between January and December 2021.

The orange line indicates the WPAI-GH score of the reference group, defined as participants without a record of health insurance claim issuance (i.e., those who had no diseases) between January and December 2021.
Diseases recorded in <10 participants are not plotted in the diagrams.

# **Supplementary Figure 3. Percentage of participants with a WPAI-GH score of >0% in terms of absenteeism (A), presenteeism (B), total work productivity impairment (C), and total activity impairment (D) by the period prevalence of diseases (definition 2: diseases and conditions of interests) among male workers of ≥50 years old**


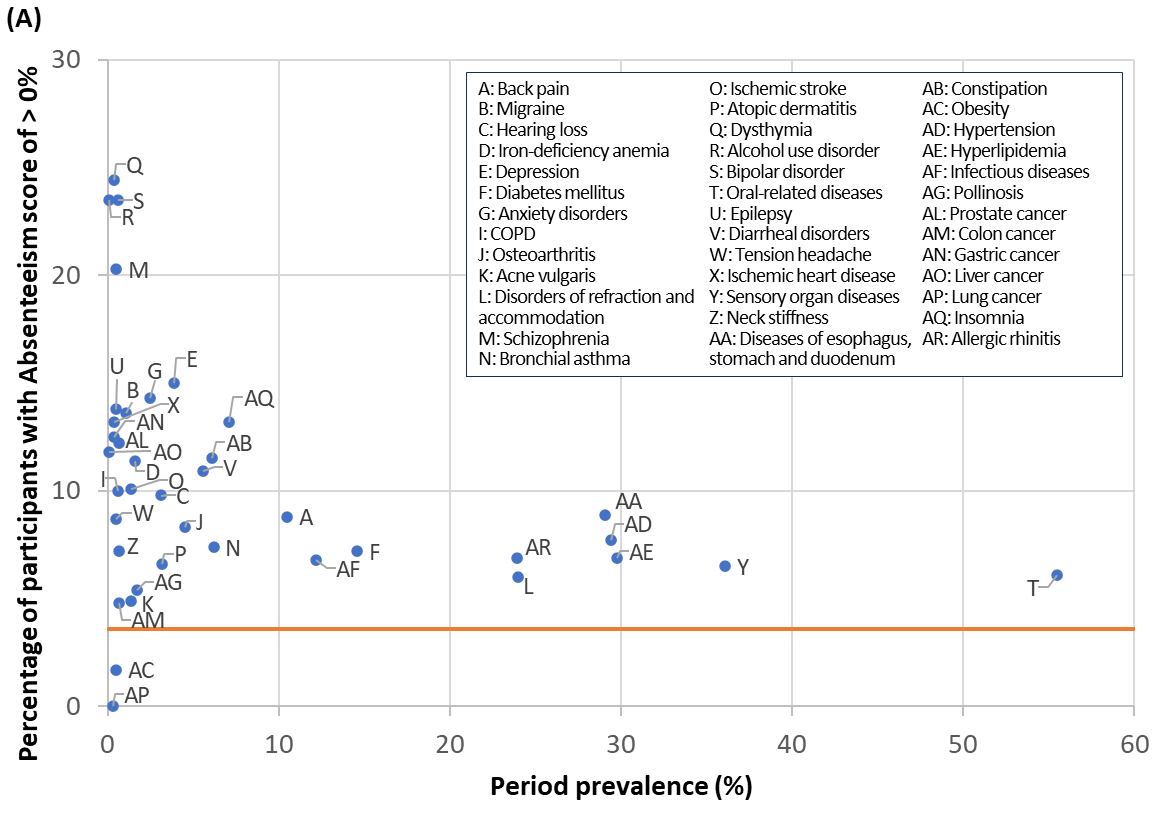


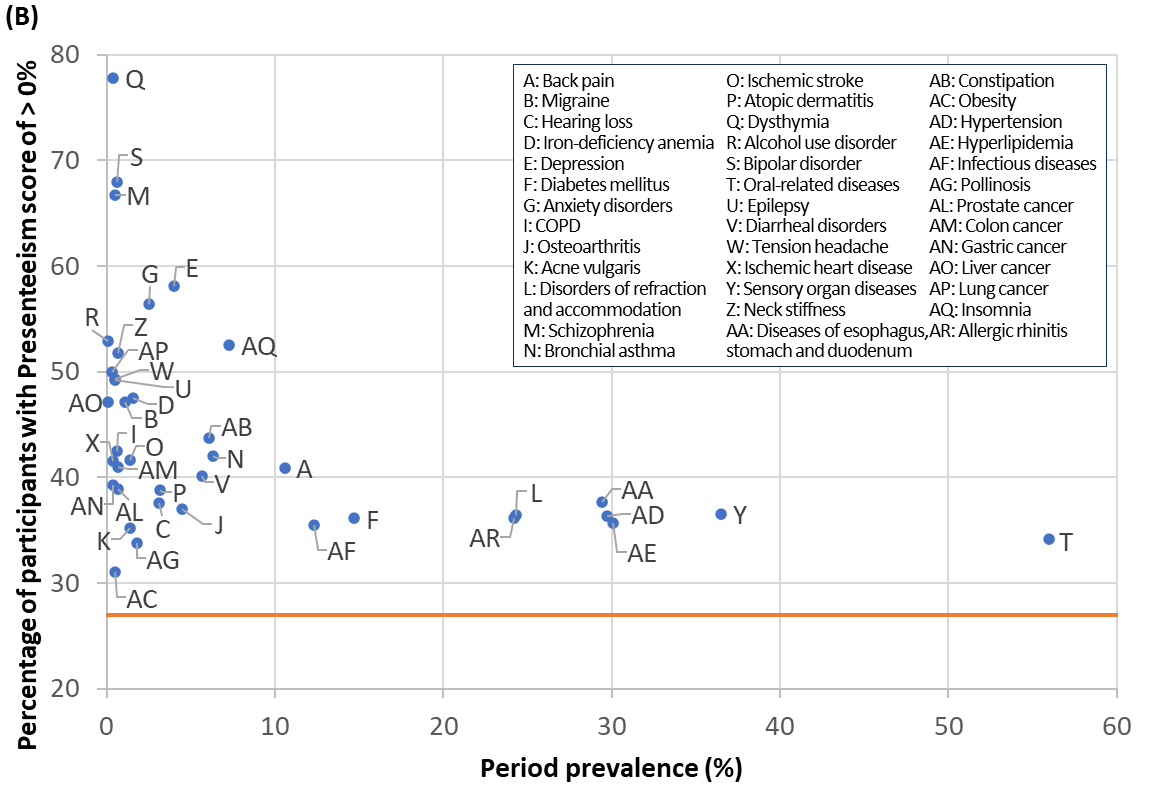

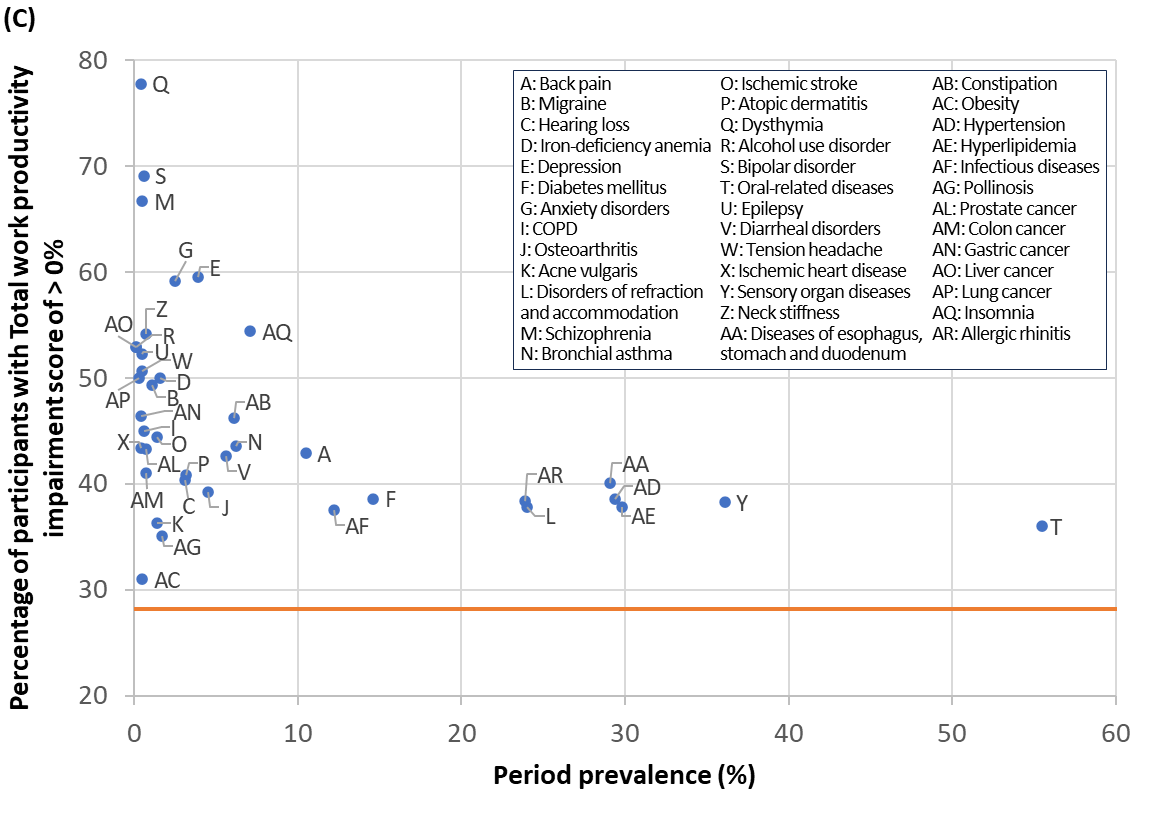


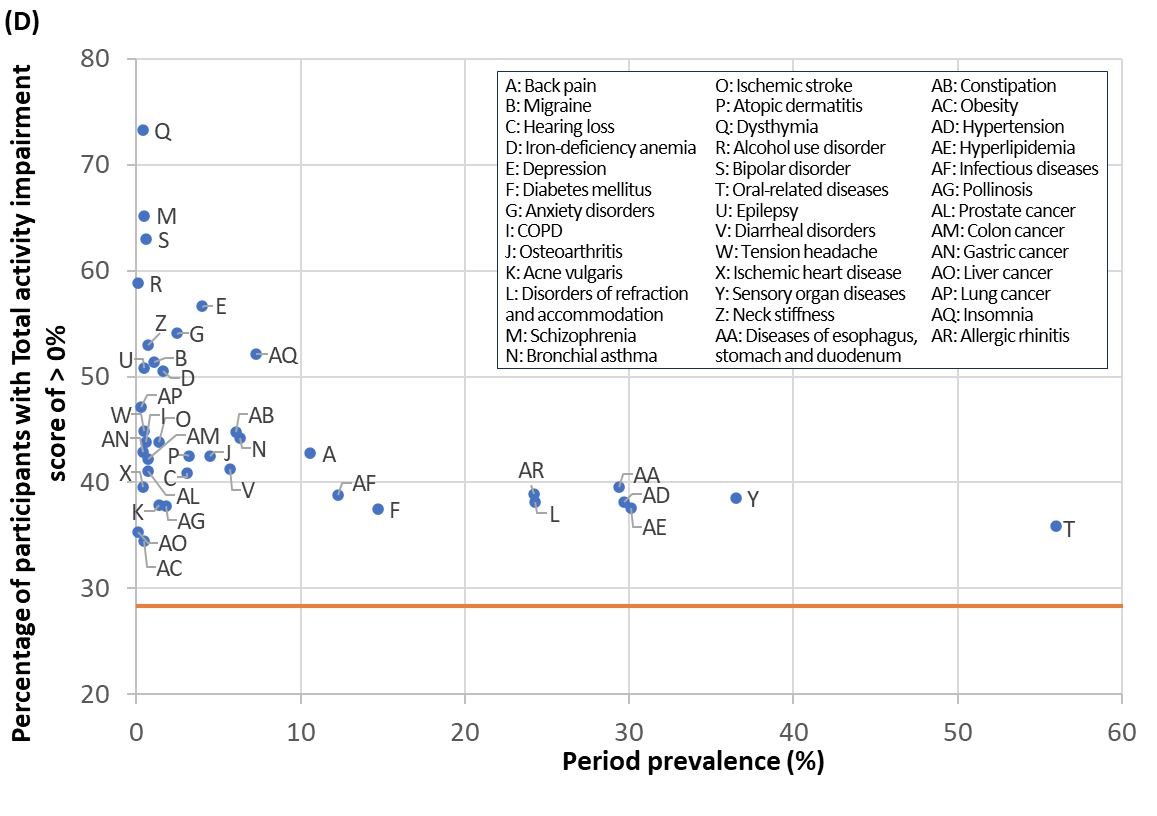


WPAI-GH, Work Productivity and Activity Impairment questionnaire-General Health

The period prevalence was calculated as the proportion of participants with a record of a specific disease more than once between January and December 2021.

The orange line indicates the WPAI-GH score of the reference group, defined as participants without a record of health insurance claim issuance (i.e., those who had no diseases) between January and December 2021.
Diseases recorded in <10 participants are not plotted in the diagrams.

# **Supplementary Figure 4. Percentage of participants with a WPAI-GH score of >0% in terms of absenteeism (A), presenteeism (B), total work productivity impairment (C), and total activity impairment (D) by the period prevalence of diseases (definition 2: diseases and conditions of interests) among female workers of ≤29 years old**


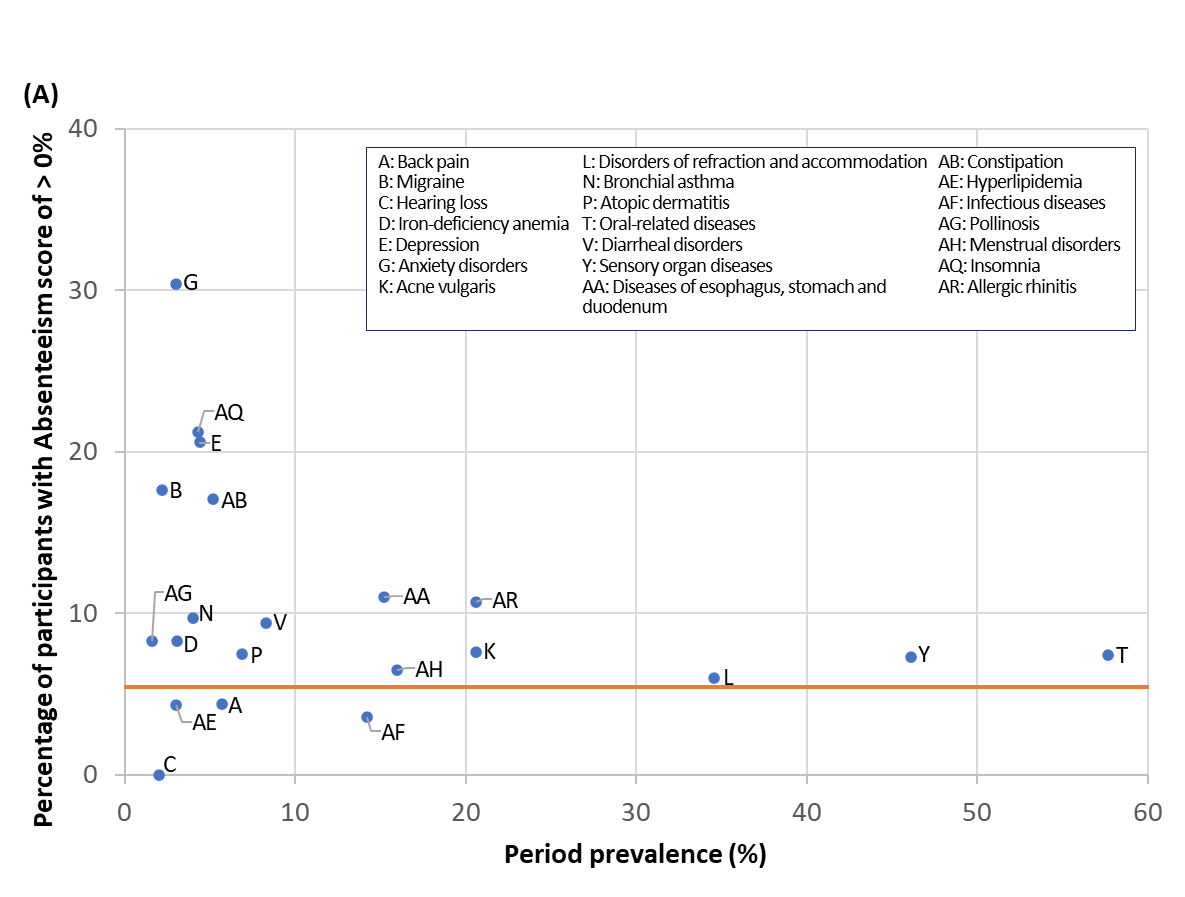

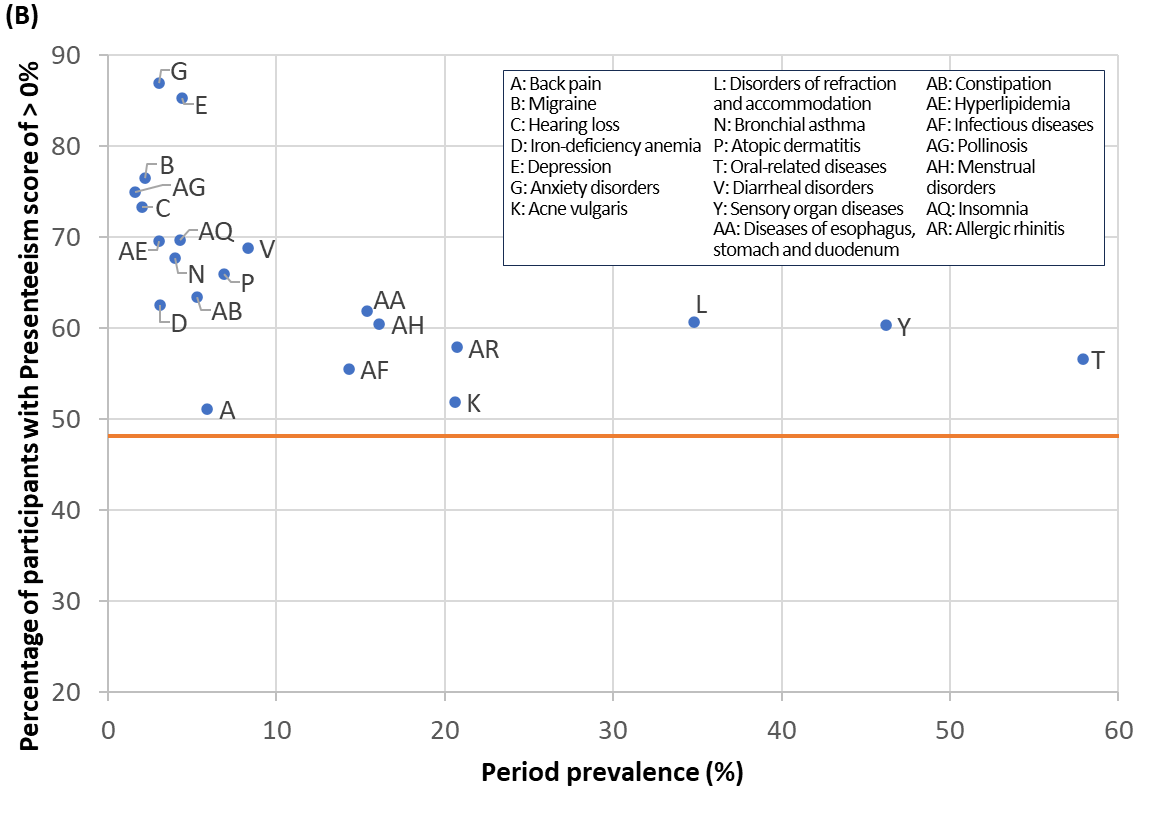


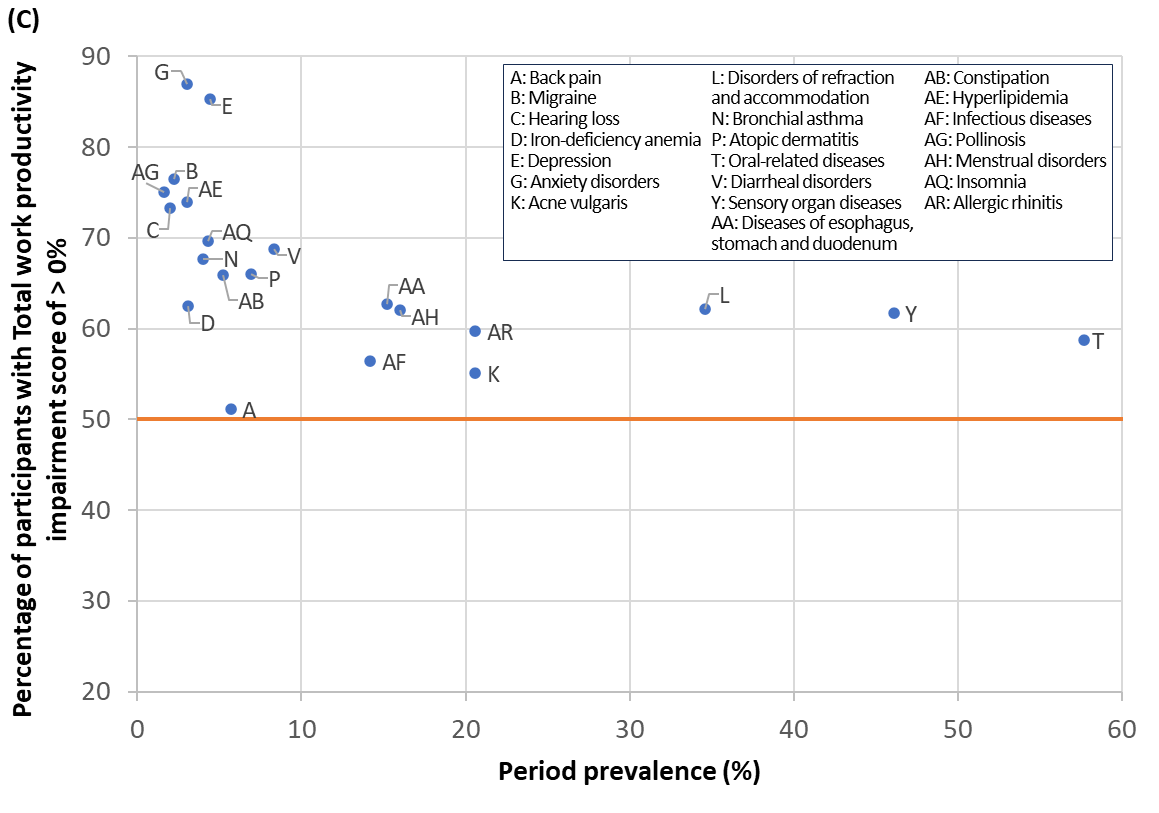


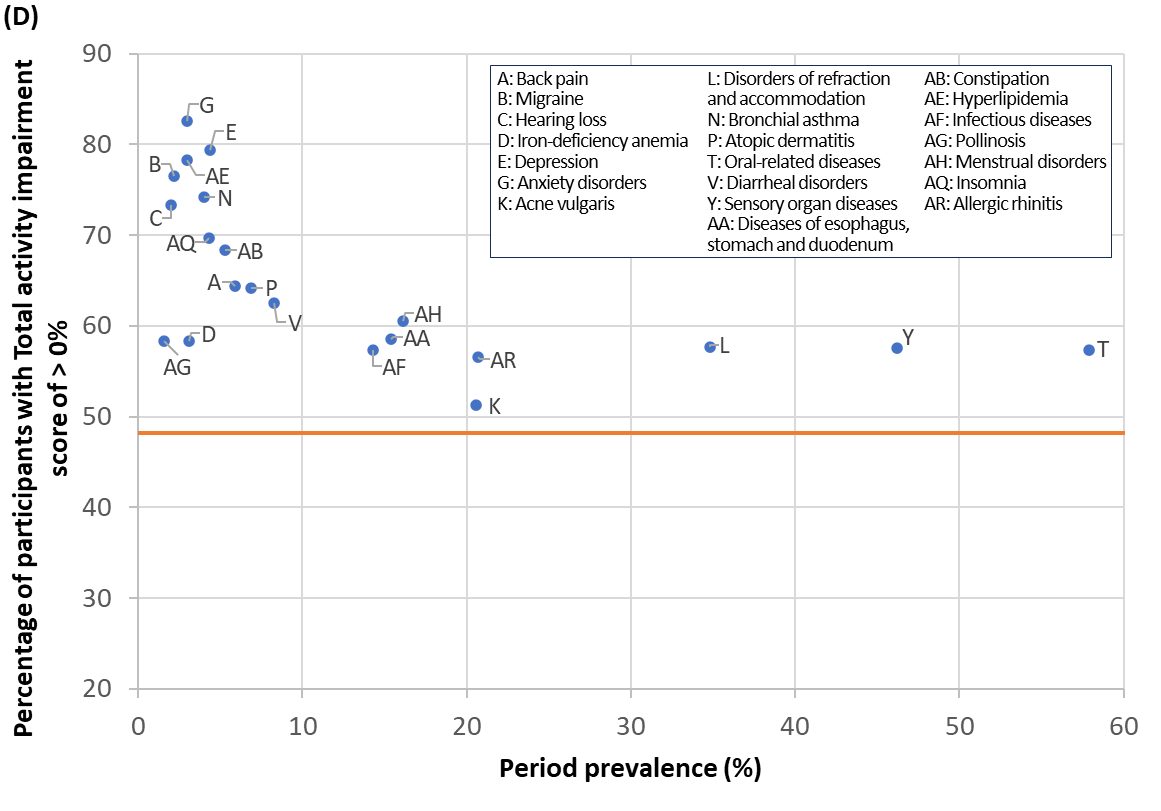


WPAI-GH, Work Productivity and Activity Impairment questionnaire-General Health

The period prevalence was calculated as the proportion of participants with a record of a specific disease more than once between January and December 2021.

The orange line indicates the WPAI-GH score of the reference group, defined as participants without a record of health insurance claim issuance (i.e., those who had no diseases) between January and December 2021.
Diseases recorded in <10 participants are not plotted in the diagrams.

# **Supplementary Figure 5. Percentage of participants with a WPAI-GH score of >0% in terms of absenteeism (A), presenteeism (B), total work productivity impairment (C), and total activity impairment (D) by the period prevalence of diseases (definition 2: diseases and conditions of interests) among female workers of 30–49 years old**


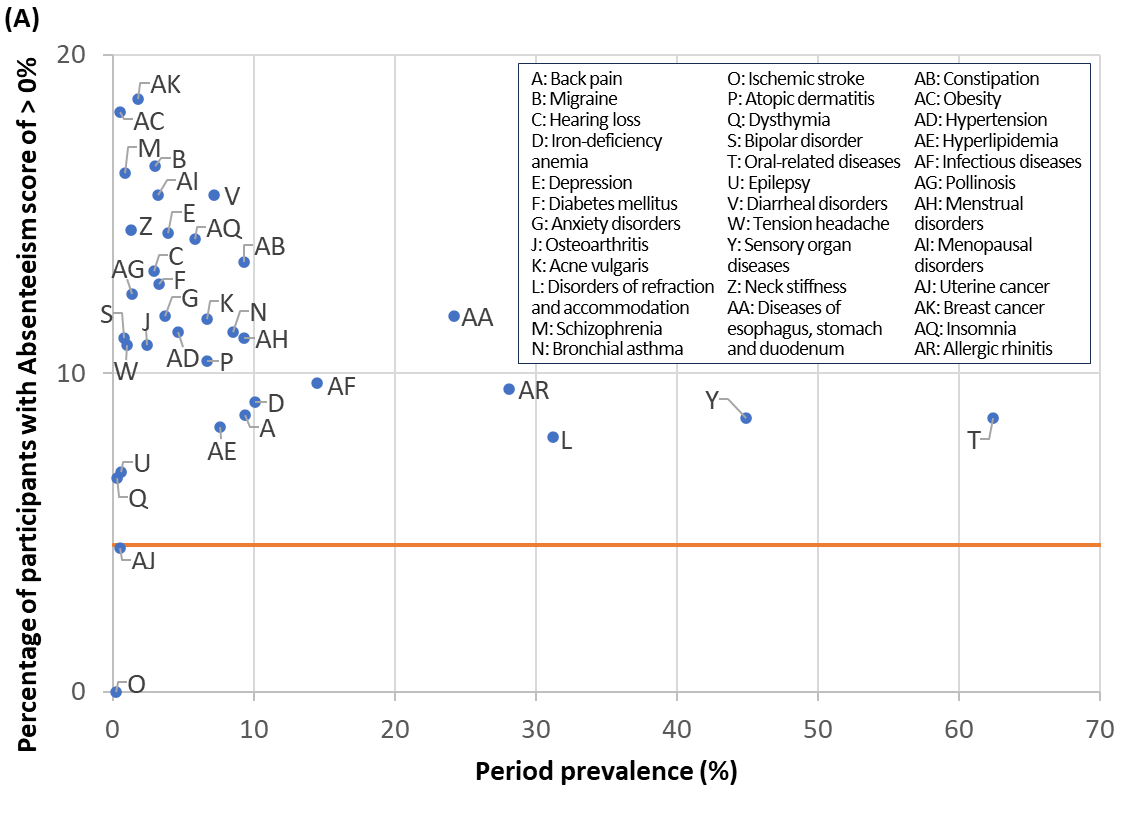


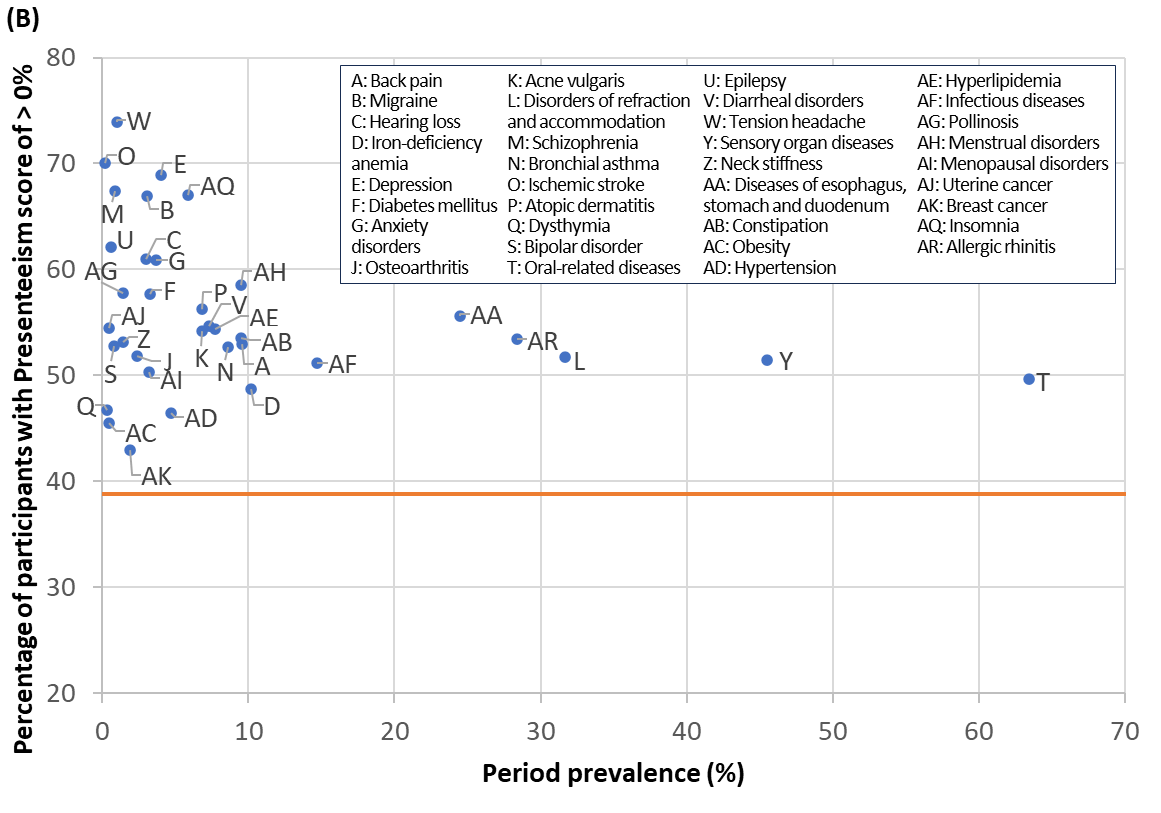


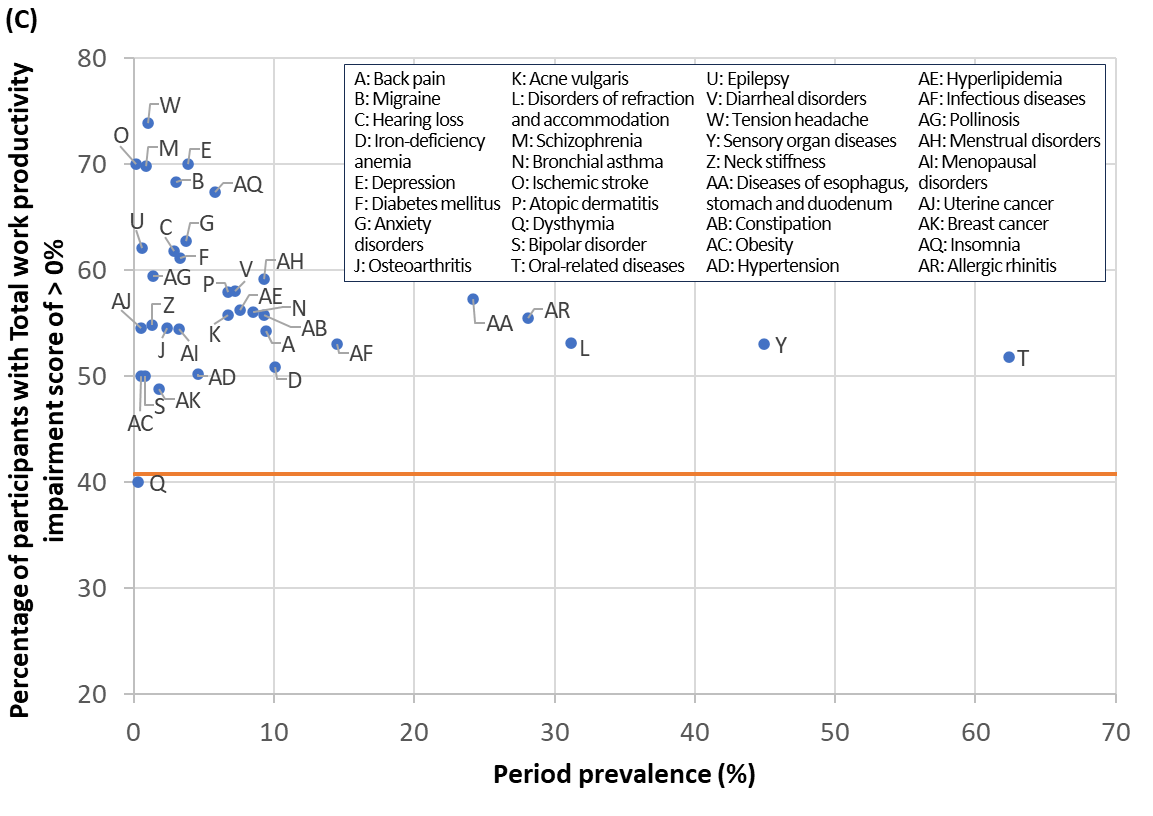


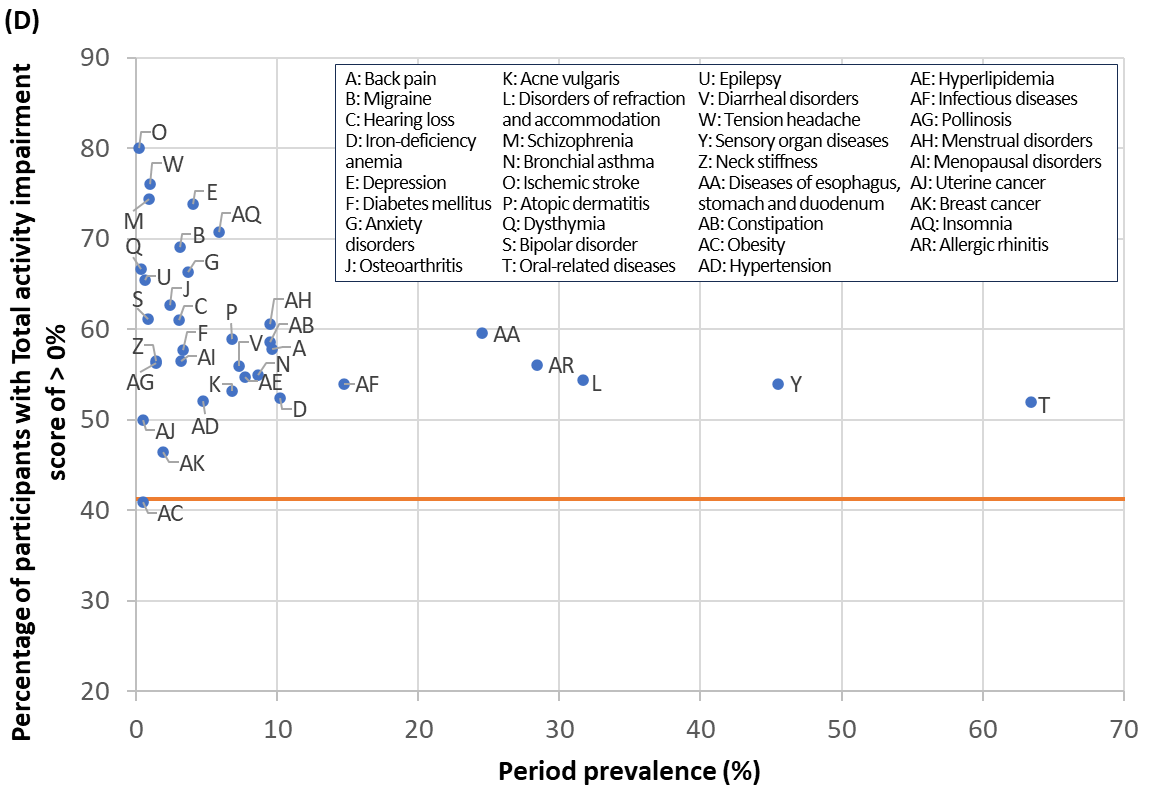


WPAI-GH, Work Productivity and Activity Impairment questionnaire-General Health

The period prevalence was calculated as the proportion of participants with a record of a specific disease more than once between January and December 2021.

The orange line indicates the WPAI-GH score of the reference group, defined as participants without a record of health insurance claim issuance (i.e., those who had no diseases) between January and December 2021.
Diseases recorded in <10 participants are not plotted in the diagrams.

# **Supplementary Figure 6. Percentage of participants with a WPAI-GH score of >0% in terms of absenteeism (A), presenteeism (B), total work productivity impairment (C), and total activity impairment (D) by the period prevalence of diseases (definition 2: diseases and conditions of interests) among female workers of ≥50 years old**


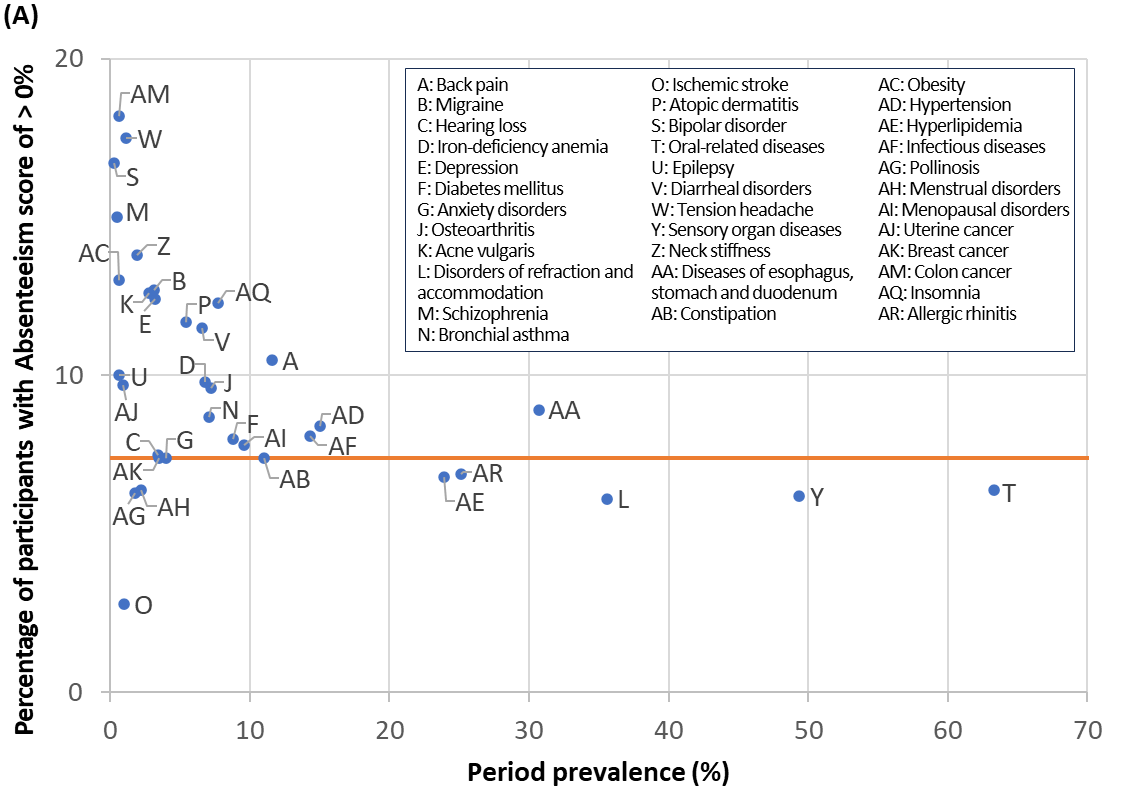


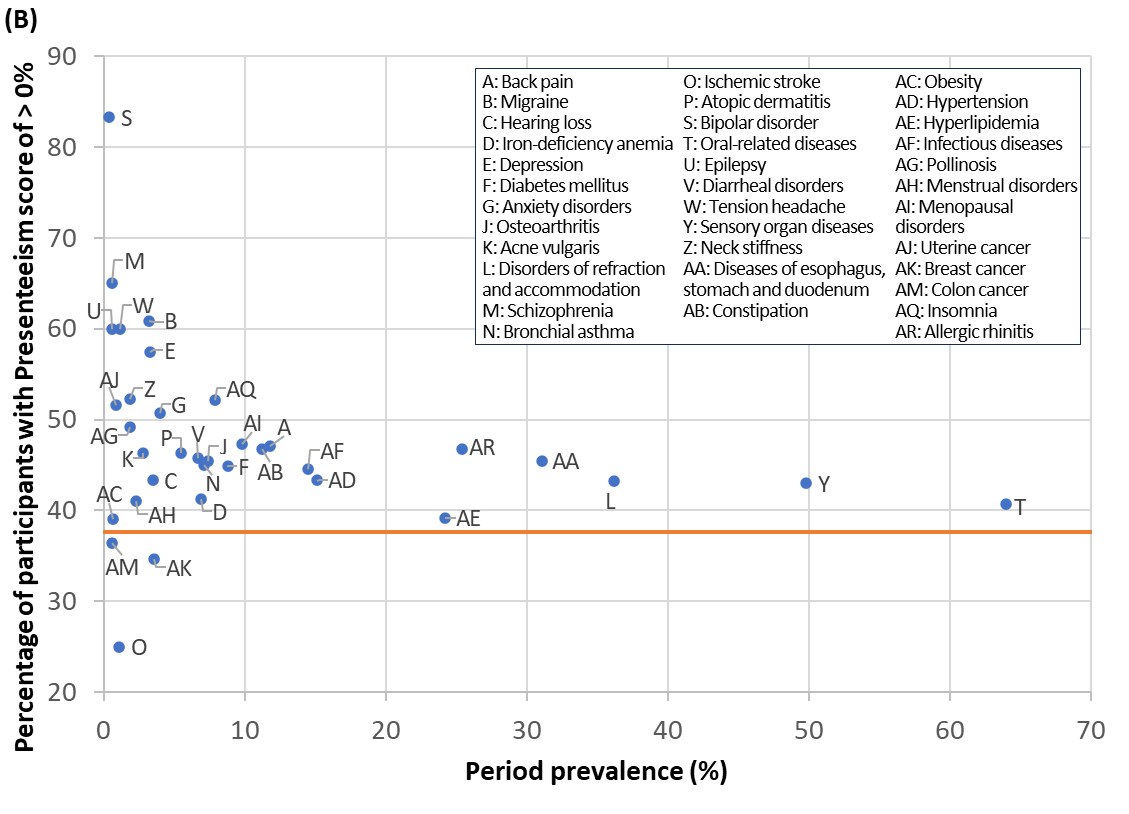


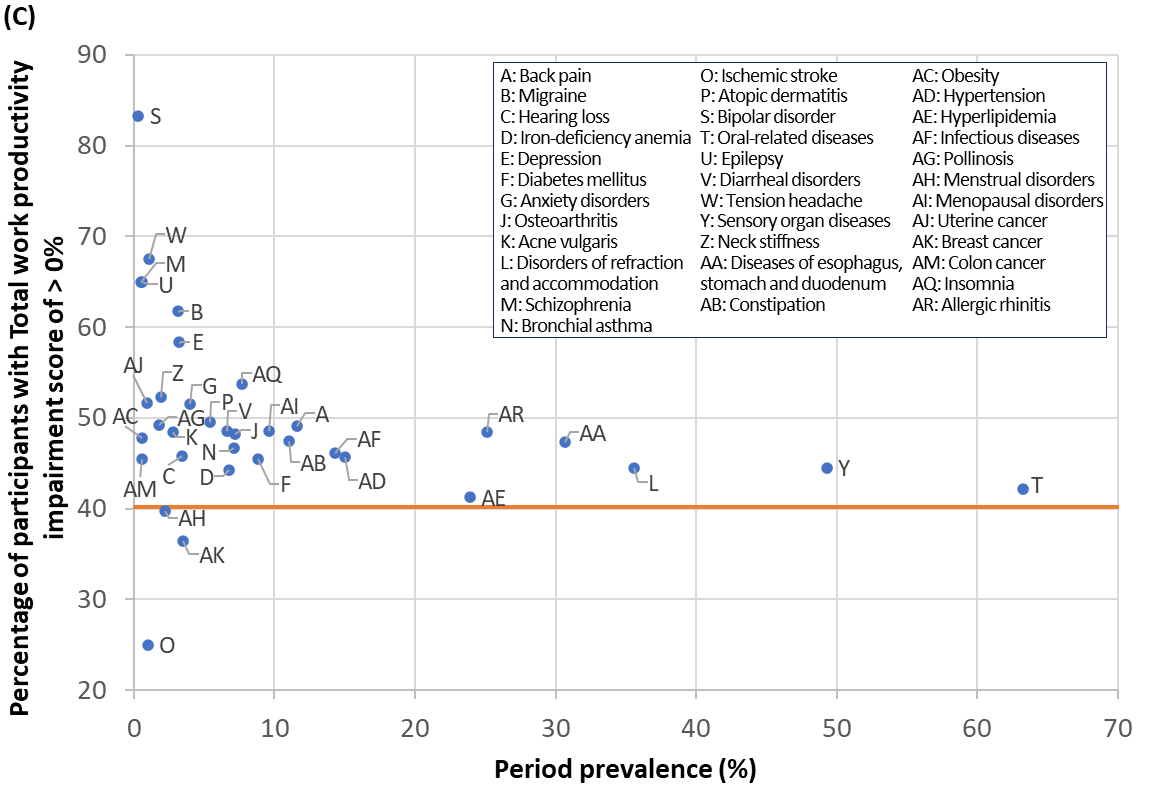


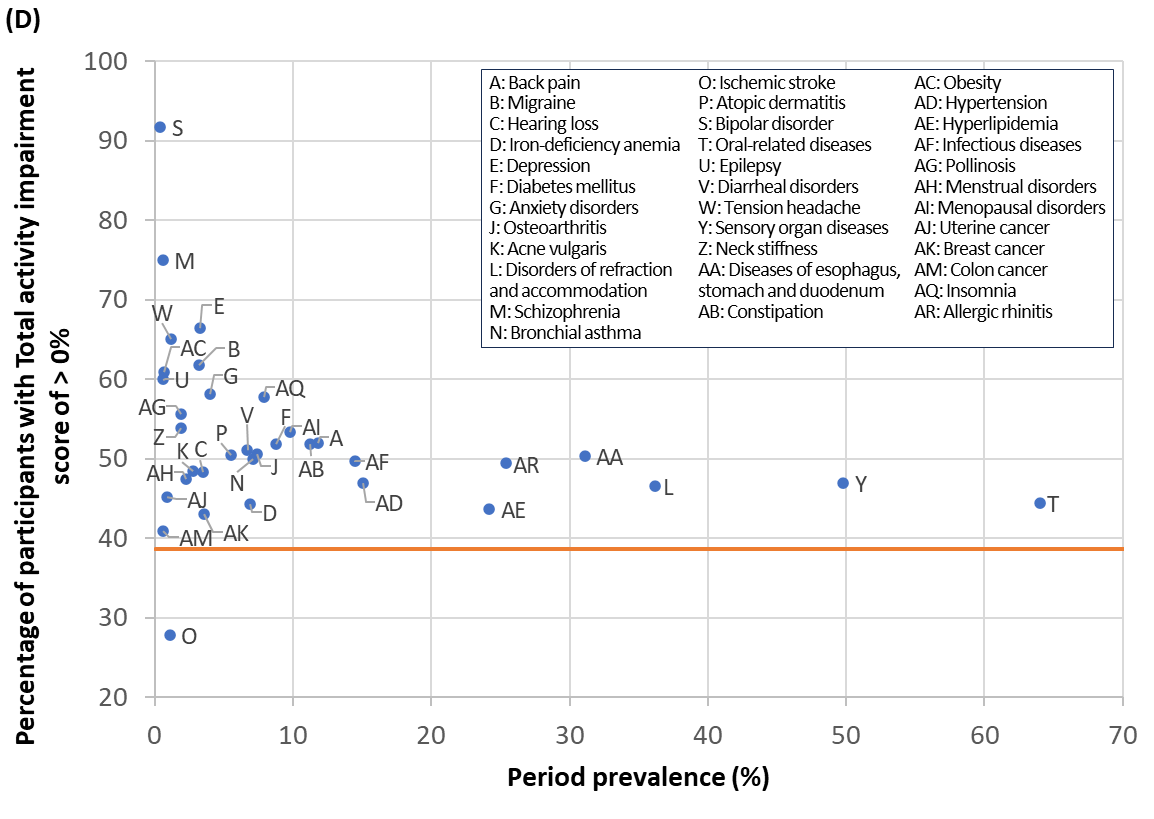


WPAI-GH, Work Productivity and Activity Impairment questionnaire-General Health

The period prevalence was calculated as the proportion of participants with a record of a specific disease more than once between January and December 2021.

The orange line indicates the WPAI-GH score of the reference group, defined as participants without a record of health insurance claim issuance (i.e., those who had no diseases) between January and December 2021.
Diseases recorded in <10 participants are not plotted in the diagrams.

# **Supplementary Table 1. Definitions of diseases**

1. Definition 1

| Disease classification (ICD-10 chapter title) | ICD-10 code |
| --- | --- |
| Certain infectious and parasitic diseases | A00 - B99 |
| Neoplasms | C00 - D48 |
| Diseases of the blood and blood-forming organs and certain disorders involving the immune mechanism | D50 - D89 |
| Endocrine, nutritional and metabolic diseases | E00 - E90 |
| Mental and behavioural disorders | F00 - F99 |
| Diseases of the nervous system | G00 - G99 |
| Diseases of the eye and adnexa | H00 - H59 |
| Diseases of the ear and mastoid process | H60 - H95 |
| Diseases of the circulatory system | I00 - I99 |
| Diseases of the respiratory system | J00 - J99 |
| Diseases of the digestive system | K00 - K93 |
| Diseases of the skin and subcutaneous tissue | L00 - L99 |
| Diseases of the musculoskeletal system and connective tissue | M00 - M99 |
| Diseases of the genitourinary system | N00 - N99 |
| Obstetric and gynecological diseases | O00 - O99 |
| Certain conditions originating in the perinatal period | P00 - P96 |
| Congenital malformations, deformations and chromosomal abnormalities | Q00 - Q99 |
| Symptoms, signs and abnormal clinical and laboratory findings, not elsewhere classified | R00 - R99 |
| Injury, poisoning and certain other consequences of external causes | S00 - T98 |
| Codes for special purposes | U00 - U89 |
| External causes of morbidity and mortality | V01 - Y98 |
| Factors influencing health status and contact with health services | Z00 - Z99 |

(B) Definition 2

| Diseases of interest | ICD-10 code |
| --- | --- |
| Back pain | M54^7^ |
| Migraine | G43^8^ |
| Hearing loss | H90, H91^9^ |
| Iron-deficiency anemia | D50.0, D50.1, D50.8, D50.9^10^ |
| Depression | F32 - F33^7^ |
| Diabetes mellitus | E10 - E14^11^ |
| Anxiety disorders | F40 - F42^12^ |
| Femoral neck fracture | S72.0^13^ |
| COPD | J43.1, J43.2, J43.8, J43.9, J44^7^ |
| Osteoarthritis | M15 - M17^14^ |
| Acne vulgaris | L70.0, L70.1, L70.8, L70.9^15^ |
| Disorders of refraction and accommodation | H52 |
| Schizophrenia | F20^16^ |
| Bronchial asthma | J45, J46^7^ |
| Ischemic stroke | I63.0, I63.1, I63.2, I63.3, I63.4, I63.5, I63.6, I63.8, I63.9, G45.9^17^ |
| Atopic dermatitis | L20 |
| Dysthymia | F34.1 |
| Alcohol use disorder | F10.2^18^ |
| Bipolar disorder | F30, F31^19^ |
| Oral-related diseases | K00 - K14^20^ |
| Epilepsy | G40.1, G40.2, G40.9^21^ |
| Diarrheal disorders | A00 - A09, K58.0, K59.1, P78.3 |
| Tension headache | G44.2^22^ |
| Ischemic heart disease | I21.0, I21.1, I21.2, I21.3, I21.4, I21.9^23^ |
| Sensory organ diseases | H00 - H95 |
| Neck stiffness | M62.81 |
| Diseases of esophagus, stomach and duodenum | K20 - K31 |
| Constipation | K59.0^24^ |
| Obesity | E66 |
| Hypertension | I10 - I15^11^ |
| Hyperlipidemia | E78^11^ |
| Infectious diseases | A15 - A99, B00 - B49 |
| Pollinosis | J30.1^25^ |
| Menstrual disorders | N80^26^, N94.3, N94.4, N94.5, N94.6 |
| Menopausal disorders | N95^27^ |
| Uterine cancer | C53 - C55^28^ |
| Breast cancer | C50^28^ |
| Prostate cancer | C61^28^ |
| Colon cancer | C18 - C20^28^ |
| Gastric cancer | C16^28^ |
| Liver cancer | C22^28^ |
| Lung cancer | C33, C34^28^ |
| Insomnia | G47.0 |
| Allergic rhinitis | J30.1, J30.2, J30.3, J30.4 |

ICD-10, International Classification of Diseases, 10th Revision

Diseases annotated with reference citations were selected based on the cited reference. Otherwise, the diseases were selected based on the clinical discretion of the study investigators.

# **Supplementary Table 2. Percentage of participants with a** **WPAI-GH score of >0% in terms of absenteeism (A), presenteeism (B), total work productivity impairment (C), and total activity impairment (D) by the period prevalence of diseases (definition 1: disease classification) among male workers**

A) Absenteeism

| **Disease^a^** | **N with disease and score calculable^b^ (A)** | **Period prevalence of disease^c^ (%)** | **N with score not calculable^b^ (B)** | **N with score >0% (C)** | **% with score >0% (C/A+B)** |
| --- | --- | --- | --- | --- | --- |
| Reference | 2,463 | – | 24 | 88 | 3.5 |
| A00-B99 | 3,766 | 16.5 | 32 | 341 | 9.0 |
| C00-D48 | 1,661 | 7.3 | 18 | 162 | 9.6 |
| D50-D89 | 742 | 3.3 | 8 | 83 | 11.1 |
| E00-E90 | 6,827 | 29.9 | 75 | 548 | 7.9 |
| F00-F99 | 2,194 | 9.6 | 19 | 289 | 13.1 |
| G00-G99 | 3,807 | 16.7 | 40 | 390 | 10.1 |
| H00-H59 | 6,764 | 29.6 | 62 | 454 | 6.7 |
| H60-H95 | 1,543 | 6.8 | 15 | 134 | 8.6 |
| I00-I99 | 5,650 | 24.8 | 55 | 453 | 7.9 |
| J00-J99 | 7,826 | 34.3 | 72 | 602 | 7.6 |
| K00-K93 | 15,493 | 67.9 | 156 | 1,045 | 6.7 |
| L00-L99 | 5,370 | 23.5 | 42 | 418 | 7.7 |
| M00-M99 | 6,098 | 26.7 | 57 | 526 | 8.5 |
| N00-N99 | 2,510 | 11.0 | 26 | 224 | 8.8 |
| O00-O99 | 1 | 0 | 0 | 1 | 100 |
| P00-P96 | 1 | 0 | 0 | 0 | 0 |
| Q00-Q99 | 269 | 1.2 | 1 | 25 | 9.3 |
| R00-R99 | 5,186 | 22.7 | 53 | 499 | 9.5 |
| S00-T98 | 4,494 | 19.7 | 38 | 373 | 8.2 |
| U00-U89 | 0 | – | 0 | 0 | – |
| V01-Y98 | 94 | 0.4 | 0 | 12 | 12.8 |
| Z00-Z99 | 649 | 2.8 | 9 | 68 | 10.3 |

B) Presenteeism

| **Disease^a^** | **N with disease and score calculable^b^ (A)** | **Period prevalence of disease^c^ (%)** | **N with score not calculable^b^ (B)** | **N with score >0% (C)** | **% with score >0% (C/A+B)** |
| --- | --- | --- | --- | --- | --- |
| Reference | 2,487 | – | 0 | 803 | 32.3 |
| A00-B99 | 3,798 | 16.6 | 0 | 1,538 | 40.5 |
| C00-D48 | 1,679 | 7.4 | 0 | 688 | 41.0 |
| D50-D89 | 750 | 3.3 | 0 | 346 | 46.1 |
| E00-E90 | 6,902 | 30.2 | 0 | 2,668 | 38.7 |
| F00-F99 | 2,213 | 9.7 | 0 | 1,187 | 53.6 |
| G00-G99 | 3,847 | 16.9 | 0 | 1,847 | 48.0 |
| H00-H59 | 6,826 | 29.9 | 0 | 2,704 | 39.6 |
| H60-H95 | 1,558 | 6.8 | 0 | 648 | 41.6 |
| I00-I99 | 5,705 | 25.0 | 0 | 2,186 | 38.3 |
| J00-J99 | 7,898 | 34.6 | 0 | 3,224 | 40.8 |
| K00-K93 | 15,649 | 68.6 | 0 | 6,009 | 38.4 |
| L00-L99 | 5,412 | 23.7 | 0 | 2,192 | 40.5 |
| M00-M99 | 6,155 | 27.0 | 0 | 2,556 | 41.5 |
| N00-N99 | 2,536 | 11.1 | 0 | 1,055 | 41.6 |
| O00-O99 | 1 | 0 | 0 | 1 | 100 |
| P00-P96 | 1 | 0 | 0 | 1 | 100 |
| Q00-Q99 | 270 | 1.2 | 0 | 109 | 40.4 |
| R00-R99 | 5,239 | 23.0 | 0 | 2,316 | 44.2 |
| S00-T98 | 4,532 | 19.9 | 0 | 1,765 | 38.9 |
| U00-U89 | 0 | – | 0 | 0 | – |
| V01-Y98 | 94 | 0.4 | 0 | 48 | 51.1 |
| Z00-Z99 | 658 | 2.9 | 0 | 265 | 40.3 |

C) Total work productivity impairment

| **Disease^a^** | **N with disease and score calculable^b^ (A)** | **Period prevalence of disease^c^ (%)** | **N with score not calculable^b^ (B)** | **N with score >0% (C)** | **% with score >0% (C/A+B)** |
| --- | --- | --- | --- | --- | --- |
| Reference | 2,463 | – | 24 | 832 | 33.5 |
| A00-B99 | 3,766 | 16.5 | 32 | 1,623 | 42.7 |
| C00-D48 | 1,661 | 7.3 | 18 | 733 | 43.7 |
| D50-D89 | 742 | 3.3 | 8 | 367 | 48.9 |
| E00-E90 | 6,827 | 29.9 | 75 | 2,810 | 40.7 |
| F00-F99 | 2,194 | 9.6 | 19 | 1,227 | 55.4 |
| G00-G99 | 3,807 | 16.7 | 40 | 1,920 | 49.9 |
| H00-H59 | 6,764 | 29.6 | 62 | 2,817 | 41.3 |
| H60-H95 | 1,543 | 6.8 | 15 | 683 | 43.8 |
| I00-I99 | 5,650 | 24.8 | 55 | 2,302 | 40.4 |
| J00-J99 | 7,826 | 34.3 | 72 | 3,379 | 42.8 |
| K00-K93 | 15,493 | 67.9 | 156 | 6,276 | 40.1 |
| L00-L99 | 5,370 | 23.5 | 42 | 2,308 | 42.6 |
| M00-M99 | 6,098 | 26.7 | 57 | 2,681 | 43.6 |
| N00-N99 | 2,510 | 11.0 | 26 | 1,113 | 43.9 |
| O00-O99 | 1 | 0 | 0 | 1 | 100 |
| P00-P96 | 1 | 0 | 0 | 1 | 100 |
| Q00-Q99 | 269 | 1.2 | 1 | 118 | 43.7 |
| R00-R99 | 5,186 | 22.7 | 53 | 2,418 | 46.2 |
| S00-T98 | 4,494 | 19.7 | 38 | 1,860 | 41.0 |
| U00-U89 | 0 | – | 0 | 0 | – |
| V01-Y98 | 94 | 0.4 | 0 | 51 | 54.3 |
| Z00-Z99 | 649 | 2.8 | 9 | 280 | 42.6 |

D)Total activity impairment

| **Disease^a^** | **N with disease and score calculable^b^ (A)** | **Period prevalence of disease^c^ (%)** | **N with score not calculable^b^ (B)** | **N with score >0% (C)** | **% with score >0% (C/A+B)** |
| --- | --- | --- | --- | --- | --- |
| Reference | 2,487 | – | 0 | 813 | 32.7 |
| A00-B99 | 3,798 | 16.6 | 0 | 1,588 | 41.8 |
| C00-D48 | 1,679 | 7.4 | 0 | 711 | 42.3 |
| D50-D89 | 750 | 3.3 | 0 | 349 | 46.5 |
| E00-E90 | 6,902 | 30.2 | 0 | 2,752 | 39.9 |
| F00-F99 | 2,213 | 9.7 | 0 | 1,172 | 53.0 |
| G00-G99 | 3,847 | 16.9 | 0 | 1,902 | 49.4 |
| H00-H59 | 6,826 | 29.9 | 0 | 2,788 | 40.8 |
| H60-H95 | 1,558 | 6.8 | 0 | 666 | 42.7 |
| I00-I99 | 5,705 | 25.0 | 0 | 2,282 | 40.0 |
| J00-J99 | 7,898 | 34.6 | 0 | 3,301 | 41.8 |
| K00-K93 | 15,649 | 68.6 | 0 | 6,158 | 39.4 |
| L00-L99 | 5,412 | 23.7 | 0 | 2,267 | 41.9 |
| M00-M99 | 6,155 | 27.0 | 0 | 2,694 | 43.8 |
| N00-N99 | 2,536 | 11.1 | 0 | 1,093 | 43.1 |
| O00-O99 | 1 | 0 | 0 | 1 | 100 |
| P00-P96 | 1 | 0 | 0 | 1 | 100 |
| Q00-Q99 | 270 | 1.2 | 0 | 107 | 39.6 |
| R00-R99 | 5,239 | 23.0 | 0 | 2,388 | 45.6 |
| S00-T98 | 4,532 | 19.9 | 0 | 1,875 | 41.4 |
| U00-U89 | 0 | – | 0 | 0 | – |
| V01-Y98 | 94 | 0.4 | 0 | 49 | 52.1 |
| Z00-Z99 | 658 | 2.9 | 0 | 273 | 41.5 |

^a^ Disease classification corresponding to each code is listed in Supplementary Table 1.

^b^ The score was not calculable when both working and absentee hours were 0 in the past 7 days, i.e., the denominator for calculating absenteeism and total work productivity impairment scores was zero.

^c^ A proportion of respondents with the disease and WPAI score calculable.

# **Supplementary Table 3. Percentage of participants with a WPAI-GH score of >0% in terms of absenteeism (A), presenteeism (B), total work productivity impairment (C), and total activity impairment (D) by the period prevalence of diseases (definition 1: disease classification) among female workers**

A) Absenteeism

| **Disease^a^** | **N with disease and score calculable^b^ (A)** | **Period prevalence of disease^c^ (%)** | **N with score not calculable^b^ (B)** | **N with score >0% (C)** | **% with score >0% (C/A+B)** |
| --- | --- | --- | --- | --- | --- |
| Reference | 498 | – | 7 | 29 | 5.7 |
| A00-B99 | 1,769 | 20.3 | 25 | 175 | 9.8 |
| C00-D48 | 1,976 | 22.7 | 31 | 184 | 9.2 |
| D50-D89 | 993 | 11.4 | 11 | 98 | 9.8 |
| E00-E90 | 2,388 | 27.4 | 34 | 209 | 8.6 |
| F00-F99 | 1,069 | 12.3 | 20 | 116 | 10.7 |
| G00-G99 | 1,416 | 16.2 | 24 | 170 | 11.8 |
| H00-H59 | 3,720 | 42.7 | 47 | 265 | 7.0 |
| H60-H95 | 789 | 9.1 | 13 | 79 | 9.9 |
| I00-I99 | 1,307 | 15.0 | 15 | 120 | 9.1 |
| J00-J99 | 3,241 | 37.2 | 38 | 293 | 8.9 |
| K00-K93 | 6,410 | 73.6 | 79 | 505 | 7.8 |
| L00-L99 | 3,282 | 37.7 | 40 | 307 | 9.2 |
| M00-M99 | 2,563 | 29.4 | 34 | 238 | 9.2 |
| N00-N99 | 2,868 | 32.9 | 39 | 260 | 8.9 |
| O00-O99 | 187 | 2.1 | 6 | 32 | 16.6 |
| P00-P96 | 5 | 0.1 | 1 | 0 | 0 |
| Q00-Q99 | 162 | 1.9 | 5 | 13 | 7.8 |
| R00-R99 | 2,536 | 29.1 | 35 | 274 | 10.7 |
| S00-T98 | 1,880 | 21.6 | 25 | 170 | 8.9 |
| U00-U89 | 0 | – | 0 | 0 | – |
| V01-Y98 | 32 | 0.4 | 0 | 8 | 25.0 |
| Z00-Z99 | 194 | 2.2 | 4 | 23 | 11.6 |

B) Presenteeism

| **Disease^a^** | **N with disease and score calculable^b^ (A)** | **Period prevalence of disease^c^ (%)** | **N with score not calculable^b^ (B)** | **N with score >0% (C)** | **% with score >0% (C/A+B)** |
| --- | --- | --- | --- | --- | --- |
| Reference | 505 | – | 0 | 199 | 39.4 |
| A00-B99 | 1,794 | 20.6 | 0 | 888 | 49.5 |
| C00-D48 | 2,007 | 23.0 | 0 | 987 | 49.2 |
| D50-D89 | 1,004 | 11.5 | 0 | 483 | 48.1 |
| E00-E90 | 2,422 | 27.8 | 0 | 1,167 | 48.2 |
| F00-F99 | 1,089 | 12.5 | 0 | 625 | 57.4 |
| G00-G99 | 1,440 | 16.5 | 0 | 810 | 56.3 |
| H00-H59 | 3,767 | 43.2 | 0 | 1,825 | 48.4 |
| H60-H95 | 802 | 9.2 | 0 | 427 | 53.2 |
| I00-I99 | 1,322 | 15.2 | 0 | 616 | 46.6 |
| J00-J99 | 3,279 | 37.6 | 0 | 1,659 | 50.6 |
| K00-K93 | 6,489 | 74.5 | 0 | 3,068 | 47.3 |
| L00-L99 | 3,322 | 38.1 | 0 | 1,656 | 49.8 |
| M00-M99 | 2,597 | 29.8 | 0 | 1,297 | 49.9 |
| N00-N99 | 2,907 | 33.4 | 0 | 1,448 | 49.8 |
| O00-O99 | 193 | 2.2 | 0 | 100 | 51.8 |
| P00-P96 | 6 | 0.1 | 0 | 3 | 50.0 |
| Q00-Q99 | 167 | 1.9 | 0 | 72 | 43.1 |
| R00-R99 | 2,571 | 29.5 | 0 | 1,321 | 51.4 |
| S00-T98 | 1,905 | 21.9 | 0 | 886 | 46.5 |
| U00-U89 | 0 | – | 0 | 0 | – |
| V01-Y98 | 32 | 0.4 | 0 | 15 | 46.9 |
| Z00-Z99 | 198 | 2.3 | 0 | 101 | 51.0 |

C) Total work productivity impairment

| **Disease^a^** | **N with disease and score calculable^b^ (A)** | **Period prevalence of disease^c^ (%)** | **N with score not calculable^b^ (B)** | **N with score >0% (C)** | **% with score >0% (C/A+B)** |
| --- | --- | --- | --- | --- | --- |
| Reference | 498 | – | 7 | 210 | 41.6 |
| A00-B99 | 1,769 | 20.3 | 25 | 922 | 51.4 |
| C00-D48 | 1,976 | 22.7 | 31 | 1,026 | 51.1 |
| D50-D89 | 993 | 11.4 | 11 | 508 | 50.6 |
| E00-E90 | 2,388 | 27.4 | 34 | 1,222 | 50.5 |
| F00-F99 | 1,069 | 12.3 | 20 | 640 | 58.8 |
| G00-G99 | 1,416 | 16.2 | 24 | 834 | 57.9 |
| H00-H59 | 3,720 | 42.7 | 47 | 1,879 | 49.9 |
| H60-H95 | 789 | 9.1 | 13 | 441 | 55.0 |
| I00-I99 | 1,307 | 15.0 | 15 | 647 | 48.9 |
| J00-J99 | 3,241 | 37.2 | 38 | 1,723 | 52.5 |
| K00-K93 | 6,410 | 73.6 | 79 | 3,187 | 49.1 |
| L00-L99 | 3,282 | 37.7 | 40 | 1,724 | 51.9 |
| M00-M99 | 2,563 | 29.4 | 34 | 1,345 | 51.8 |
| N00-N99 | 2,868 | 32.9 | 39 | 1,504 | 51.7 |
| O00-O99 | 187 | 2.1 | 6 | 105 | 54.4 |
| P00-P96 | 5 | 0.1 | 1 | 3 | 50.0 |
| Q00-Q99 | 162 | 1.9 | 5 | 73 | 43.7 |
| R00-R99 | 2,536 | 29.1 | 35 | 1,379 | 53.6 |
| S00-T98 | 1,880 | 21.6 | 25 | 921 | 48.3 |
| U00-U89 | 0 | – | 0 | 0 | – |
| V01-Y98 | 32 | 0.4 | 0 | 18 | 56.3 |
| Z00-Z99 | 194 | 2.2 | 4 | 108 | 54.5 |

D)Total activity impairment

| **Disease^a^** | **N with disease and score calculable^b^ (A)** | **Period prevalence of disease^c^ (%)** | **N with score not calculable^b^ (B)** | **N with score >0% (C)** | **% with score >0% (C/A+B)** |
| --- | --- | --- | --- | --- | --- |
| Reference | 505 | – | 0 | 207 | 41.0 |
| A00-B99 | 1,794 | 20.6 | 0 | 949 | 52.9 |
| C00-D48 | 2,007 | 23.0 | 0 | 1,064 | 53.0 |
| D50-D89 | 1,004 | 11.5 | 0 | 516 | 51.4 |
| E00-E90 | 2,422 | 27.8 | 0 | 1,256 | 51.9 |
| F00-F99 | 1,089 | 12.5 | 0 | 661 | 60.7 |
| G00-G99 | 1,440 | 16.5 | 0 | 867 | 60.2 |
| H00-H59 | 3,767 | 43.2 | 0 | 1,918 | 50.9 |
| H60-H95 | 802 | 9.2 | 0 | 460 | 57.4 |
| I00-I99 | 1,322 | 15.2 | 0 | 670 | 50.7 |
| J00-J99 | 3,279 | 37.6 | 0 | 1,745 | 53.2 |
| K00-K93 | 6,489 | 74.5 | 0 | 3,261 | 50.3 |
| L00-L99 | 3,322 | 38.1 | 0 | 1,741 | 52.4 |
| M00-M99 | 2,597 | 29.8 | 0 | 1,405 | 54.1 |
| N00-N99 | 2,907 | 33.4 | 0 | 1,548 | 53.3 |
| O00-O99 | 193 | 2.2 | 0 | 111 | 57.5 |
| P00-P96 | 6 | 0.1 | 0 | 3 | 50.0 |
| Q00-Q99 | 167 | 1.9 | 0 | 79 | 47.3 |
| R00-R99 | 2,571 | 29.5 | 0 | 1,406 | 54.7 |
| S00-T98 | 1,905 | 21.9 | 0 | 962 | 50.5 |
| U00-U89 | 0 | – | 0 | 0 | – |
| V01-Y98 | 32 | 0.4 | 0 | 16 | 50.0 |
| Z00-Z99 | 198 | 2.3 | 0 | 104 | 52.5 |

^a^ Disease classification corresponding to each code is listed in Supplementary Table 1.

^b^ The score was not calculable when both working and absentee hours were 0 in the past 7 days, i.e., the denominator for calculating absenteeism and total work productivity impairment scores was zero.

^c^ A proportion of respondents with the disease and WPAI score calculable.

# **Supplementary Table 4. Percentage of participants with a WPAI-GH score of >0% in terms of absenteeism (A), presenteeism (B), total work productivity impairment (C), and total activity impairment (D) by the period prevalence of diseases (definition 2: diseases and conditions of interests) among male workers**

A) Absenteeism

| **Disease^a^** | **N with disease and score calculable^b^ (A)** | **Period prevalence of disease^c^ (%)** | **N with score not calculable^b^ (B)** | **N with score >0% (C)** | **% with score >0% (C/A+B)** |
| --- | --- | --- | --- | --- | --- |
| Reference | 2,463 | – | 24 | 88 | 3.5 |
| Back pain | 1,973 | 8.6 | 20 | 186 | 9.3 |
| Migraine | 277 | 1.2 | 2 | 42 | 15.1 |
| Hearing loss | 566 | 2.5 | 4 | 55 | 9.6 |
| Iron-deficiency anemia | 274 | 1.2 | 2 | 31 | 11.2 |
| Depression | 949 | 4.2 | 13 | 154 | 16.0 |
| Diabetes mellitus | 2,315 | 10.1 | 22 | 190 | 8.1 |
| Anxiety disorders | 548 | 2.4 | 2 | 73 | 13.3 |
| Femoral neck fracture | 5 | 0 | 0 | 1 | 20.0 |
| COPD | 94 | 0.4 | 0 | 10 | 10.6 |
| Osteoarthritis | 657 | 2.9 | 4 | 55 | 8.3 |
| Acne vulgaris | 560 | 2.5 | 3 | 36 | 6.4 |
| Disorders of refraction and accommodation | 4,910 | 21.5 | 44 | 311 | 6.3 |
| Schizophrenia | 138 | 0.6 | 3 | 26 | 18.4 |
| Bronchial asthma | 1,346 | 5.9 | 16 | 109 | 8.0 |
| Ischemic stroke | 206 | 0.9 | 2 | 23 | 11.1 |
| Atopic dermatitis | 888 | 3.9 | 5 | 59 | 6.6 |
| Dysthymia | 64 | 0.3 | 0 | 14 | 21.9 |
| Alcohol use disorder | 22 | 0.1 | 0 | 6 | 27.3 |
| Bipolar disorder | 148 | 0.6 | 4 | 30 | 19.7 |
| Oral-related diseases | 12,171 | 53.3 | 121 | 781 | 6.4 |
| Epilepsy | 113 | 0.5 | 0 | 17 | 15.0 |
| Diarrheal disorders | 1,379 | 6.0 | 14 | 164 | 11.8 |
| Tension headache | 111 | 0.5 | 1 | 14 | 12.5 |
| Ischemic heart disease | 59 | 0.3 | 3 | 7 | 11.3 |
| Sensory organ diseases | 7,661 | 33.6 | 73 | 533 | 6.9 |
| Neck stiffness | 127 | 0.6 | 0 | 14 | 11.0 |
| Diseases of esophagus, stomach and duodenum | 5,503 | 24.1 | 64 | 522 | 9.4 |
| Constipation | 1,045 | 4.6 | 12 | 130 | 12.3 |
| Obesity | 91 | 0.4 | 1 | 4 | 4.3 |
| Hypertension | 4,510 | 19.8 | 45 | 357 | 7.8 |
| Hyperlipidemia | 5,012 | 22.0 | 53 | 383 | 7.6 |
| Infectious diseases | 2,506 | 11.0 | 20 | 200 | 7.9 |
| Pollinosis | 371 | 1.6 | 4 | 20 | 5.3 |
| Menstrual disorders | 0 | – | 0 | 0 | – |
| Menopausal disorders | 12 | 0.1 | 0 | 0 | 0 |
| Uterine cancer | 0 | – | 0 | 0 | – |
| Breast cancer | 2 | 0 | 0 | 0 | 0 |
| Prostate cancer | 90 | 0.4 | 1 | 11 | 12.1 |
| Colon cancer | 103 | 0.5 | 1 | 7 | 6.7 |
| Gastric cancer | 63 | 0.3 | 1 | 8 | 12.5 |
| Liver cancer | 20 | 0.1 | 0 | 3 | 15.0 |
| Lung cancer | 43 | 0.2 | 0 | 1 | 2.3 |
| Insomnia | 1,401 | 6.1 | 19 | 199 | 14.0 |
| Allergic rhinitis | 5,351 | 23.4 | 58 | 389 | 7.2 |

B) Presenteeism

| **Disease^a^** | **N with disease and score calculable^b^ (A)** | **Period prevalence of disease^c^ (%)** | **N with score not calculable^b^ (B)** | **N with score >0% (C)** | **% with score >0% (C/A+B)** |
| --- | --- | --- | --- | --- | --- |
| Reference | 2,487 | – | 0 | 803 | 32.3 |
| Back pain | 1,993 | 8.7 | 0 | 885 | 44.4 |
| Migraine | 279 | 1.2 | 0 | 151 | 54.1 |
| Hearing loss | 570 | 2.5 | 0 | 241 | 42.3 |
| Iron-deficiency anemia | 276 | 1.2 | 0 | 130 | 47.1 |
| Depression | 962 | 4.2 | 0 | 596 | 62.0 |
| Diabetes mellitus | 2,337 | 10.2 | 0 | 889 | 38.0 |
| Anxiety disorders | 550 | 2.4 | 0 | 323 | 58.7 |
| Femoral neck fracture | 5 | 0 | 0 | 3 | 60.0 |
| COPD | 94 | 0.4 | 0 | 40 | 42.6 |
| Osteoarthritis | 661 | 2.9 | 0 | 254 | 38.4 |
| Acne vulgaris | 563 | 2.5 | 0 | 246 | 43.7 |
| Disorders of refraction and accommodation | 4,954 | 21.7 | 0 | 1,933 | 39.0 |
| Schizophrenia | 141 | 0.6 | 0 | 93 | 66.0 |
| Bronchial asthma | 1,362 | 6.0 | 0 | 617 | 45.3 |
| Ischemic stroke | 208 | 0.9 | 0 | 86 | 41.3 |
| Atopic dermatitis | 893 | 3.9 | 0 | 396 | 44.3 |
| Dysthymia | 64 | 0.3 | 0 | 48 | 75.0 |
| Alcohol use disorder | 22 | 0.1 | 0 | 11 | 50.0 |
| Bipolar disorder | 152 | 0.7 | 0 | 98 | 64.5 |
| Oral-related diseases | 12,292 | 53.9 | 0 | 4,642 | 37.8 |
| Epilepsy | 113 | 0.5 | 0 | 57 | 50.4 |
| Diarrheal disorders | 1,393 | 6.1 | 0 | 637 | 45.7 |
| Tension headache | 112 | 0.5 | 0 | 60 | 53.6 |
| Ischemic heart disease | 62 | 0.3 | 0 | 25 | 40.3 |
| Sensory organ diseases | 7,734 | 33.9 | 0 | 3,087 | 39.9 |
| Neck stiffness | 127 | 0.6 | 0 | 70 | 55.1 |
| Diseases of esophagus, stomach and duodenum | 5,567 | 24.4 | 0 | 2,315 | 41.6 |
| Constipation | 1,057 | 4.6 | 0 | 490 | 46.4 |
| Obesity | 92 | 0.4 | 0 | 31 | 33.7 |
| Hypertension | 4,555 | 20.0 | 0 | 1,708 | 37.5 |
| Hyperlipidemia | 5,065 | 22.2 | 0 | 1,923 | 38.0 |
| Infectious diseases | 2,526 | 11.1 | 0 | 969 | 38.4 |
| Pollinosis | 375 | 1.6 | 0 | 145 | 38.7 |
| Menstrual disorders | 0 | – | 0 | 0 | – |
| Menopausal disorders | 12 | 0.1 | 0 | 6 | 50.0 |
| Uterine cancer | 0 | – | 0 | 0 | – |
| Breast cancer | 2 | 0 | 0 | 2 | 100 |
| Prostate cancer | 91 | 0.4 | 0 | 35 | 38.5 |
| Colon cancer | 104 | 0.5 | 0 | 44 | 42.3 |
| Gastric cancer | 64 | 0.3 | 0 | 26 | 40.6 |
| Liver cancer | 20 | 0.1 | 0 | 9 | 45.0 |
| Lung cancer | 43 | 0.2 | 0 | 21 | 48.8 |
| Insomnia | 1,420 | 6.2 | 0 | 811 | 57.1 |
| Allergic rhinitis | 5,409 | 23.7 | 0 | 2,174 | 40.2 |

C) Total work productivity impairment

| **Disease^a^** | **N with disease and score calculable^b^ (A)** | **Period prevalence of disease^c^ (%)** | **N with score not calculable^b^ (B)** | **N with score >0% (C)** | **% with score >0% (C/A+B)** |
| --- | --- | --- | --- | --- | --- |
| Reference | 2,463 | – | 24 | 832 | 33.5 |
| Back pain | 1,973 | 8.6 | 20 | 922 | 46.3 |
| Migraine | 277 | 1.2 | 2 | 157 | 56.3 |
| Hearing loss | 566 | 2.5 | 4 | 254 | 44.6 |
| Iron-deficiency anemia | 274 | 1.2 | 2 | 139 | 50.4 |
| Depression | 949 | 4.2 | 13 | 612 | 63.6 |
| Diabetes mellitus | 2,315 | 10.1 | 22 | 948 | 40.6 |
| Anxiety disorders | 548 | 2.4 | 2 | 336 | 61.1 |
| Femoral neck fracture | 5 | 0 | 0 | 3 | 60.0 |
| COPD | 94 | 0.4 | 0 | 42 | 44.7 |
| Osteoarthritis | 657 | 2.9 | 4 | 266 | 40.2 |
| Acne vulgaris | 560 | 2.5 | 3 | 256 | 45.5 |
| Disorders of refraction and accommodation | 4,910 | 21.5 | 44 | 2,011 | 40.6 |
| Schizophrenia | 138 | 0.6 | 3 | 92 | 65.2 |
| Bronchial asthma | 1,346 | 5.9 | 16 | 637 | 46.8 |
| Ischemic stroke | 206 | 0.9 | 2 | 93 | 44.7 |
| Atopic dermatitis | 888 | 3.9 | 5 | 411 | 46.0 |
| Dysthymia | 64 | 0.3 | 0 | 48 | 75.0 |
| Alcohol use disorder | 22 | 0.1 | 0 | 11 | 50.0 |
| Bipolar disorder | 148 | 0.6 | 4 | 99 | 65.1 |
| Oral-related diseases | 12,171 | 53.3 | 121 | 4,861 | 39.5 |
| Epilepsy | 113 | 0.5 | 0 | 60 | 53.1 |
| Diarrheal disorders | 1,379 | 6.0 | 14 | 674 | 48.4 |
| Tension headache | 111 | 0.5 | 1 | 62 | 55.4 |
| Ischemic heart disease | 59 | 0.3 | 3 | 25 | 40.3 |
| Sensory organ diseases | 7,661 | 33.6 | 73 | 3,222 | 41.7 |
| Neck stiffness | 127 | 0.6 | 0 | 72 | 56.7 |
| Diseases of esophagus, stomach and duodenum | 5,503 | 24.1 | 64 | 2,432 | 43.7 |
| Constipation | 1,045 | 4.6 | 12 | 515 | 48.7 |
| Obesity | 91 | 0.4 | 1 | 30 | 32.6 |
| Hypertension | 4,510 | 19.8 | 45 | 1,803 | 39.6 |
| Hyperlipidemia | 5,012 | 22.0 | 53 | 2,019 | 39.9 |
| Infectious diseases | 2,506 | 11.0 | 20 | 1,024 | 40.5 |
| Pollinosis | 371 | 1.6 | 4 | 148 | 39.5 |
| Menstrual disorders | 0 | – | 0 | 0 | – |
| Menopausal disorders | 12 | 0.1 | 0 | 6 | 50.0 |
| Uterine cancer | 0 | – | 0 | 0 | – |
| Breast cancer | 2 | 0 | 0 | 2 | 100 |
| Prostate cancer | 90 | 0.4 | 1 | 39 | 42.9 |
| Colon cancer | 103 | 0.5 | 1 | 44 | 42.3 |
| Gastric cancer | 63 | 0.3 | 1 | 30 | 46.9 |
| Liver cancer | 20 | 0.1 | 0 | 10 | 50.0 |
| Lung cancer | 43 | 0.2 | 0 | 21 | 48.8 |
| Insomnia | 1,401 | 6.1 | 19 | 837 | 58.9 |
| Allergic rhinitis | 5,351 | 23.4 | 58 | 2,274 | 42.0 |

D)Total activity impairment

| **Disease^a^** | **N with disease and score calculable^b^ (A)** | **Period prevalence of disease^c^ (%)** | **N with score not calculable^b^ (B)** | **N with score >0% (C)** | **% with score >0% (C/A+B)** |
| --- | --- | --- | --- | --- | --- |
| Reference | 2,487 | – | 0 | 813 | 32.7 |
| Back pain | 1,993 | 8.7 | 0 | 913 | 45.8 |
| Migraine | 279 | 1.2 | 0 | 158 | 56.6 |
| Hearing loss | 570 | 2.5 | 0 | 251 | 44.0 |
| Iron-deficiency anemia | 276 | 1.2 | 0 | 134 | 48.6 |
| Depression | 962 | 4.2 | 0 | 590 | 61.3 |
| Diabetes mellitus | 2,337 | 10.2 | 0 | 916 | 39.2 |
| Anxiety disorders | 550 | 2.4 | 0 | 322 | 58.5 |
| Femoral neck fracture | 5 | 0 | 0 | 3 | 60.0 |
| COPD | 94 | 0.4 | 0 | 42 | 44.7 |
| Osteoarthritis | 661 | 2.9 | 0 | 283 | 42.8 |
| Acne vulgaris | 563 | 2.5 | 0 | 244 | 43.3 |
| Disorders of refraction and accommodation | 4,954 | 21.7 | 0 | 1,989 | 40.1 |
| Schizophrenia | 141 | 0.6 | 0 | 97 | 68.8 |
| Bronchial asthma | 1,362 | 6.0 | 0 | 631 | 46.3 |
| Ischemic stroke | 208 | 0.9 | 0 | 93 | 44.7 |
| Atopic dermatitis | 893 | 3.9 | 0 | 401 | 44.9 |
| Dysthymia | 64 | 0.3 | 0 | 46 | 71.9 |
| Alcohol use disorder | 22 | 0.1 | 0 | 12 | 54.5 |
| Bipolar disorder | 152 | 0.7 | 0 | 93 | 61.2 |
| Oral-related diseases | 12,292 | 53.9 | 0 | 4,752 | 38.7 |
| Epilepsy | 113 | 0.5 | 0 | 60 | 53.1 |
| Diarrheal disorders | 1,393 | 6.1 | 0 | 639 | 45.9 |
| Tension headache | 112 | 0.5 | 0 | 57 | 50.9 |
| Ischemic heart disease | 62 | 0.3 | 0 | 24 | 38.7 |
| Sensory organ diseases | 7,734 | 33.9 | 0 | 3,173 | 41.0 |
| Neck stiffness | 127 | 0.6 | 0 | 71 | 55.9 |
| Diseases of esophagus, stomach and duodenum | 5,567 | 24.4 | 0 | 2,379 | 42.7 |
| Constipation | 1,057 | 4.6 | 0 | 497 | 47.0 |
| Obesity | 92 | 0.4 | 0 | 32 | 34.8 |
| Hypertension | 4,555 | 20.0 | 0 | 1,773 | 38.9 |
| Hyperlipidemia | 5,065 | 22.2 | 0 | 1,989 | 39.3 |
| Infectious diseases | 2,526 | 11.1 | 0 | 1,023 | 40.5 |
| Pollinosis | 375 | 1.6 | 0 | 158 | 42.1 |
| Menstrual disorders | 0 | – | 0 | 0 | – |
| Menopausal disorders | 12 | 0.1 | 0 | 7 | 58.3 |
| Uterine cancer | 0 | – | 0 | 0 | – |
| Breast cancer | 2 | 0 | 0 | 1 | 50.0 |
| Prostate cancer | 91 | 0.4 | 0 | 37 | 40.7 |
| Colon cancer | 104 | 0.5 | 0 | 45 | 43.3 |
| Gastric cancer | 64 | 0.3 | 0 | 28 | 43.8 |
| Liver cancer | 20 | 0.1 | 0 | 7 | 35.0 |
| Lung cancer | 43 | 0.2 | 0 | 20 | 46.5 |
| Insomnia | 1,420 | 6.2 | 0 | 808 | 56.9 |
| Allergic rhinitis | 5,409 | 23.7 | 0 | 2,250 | 41.6 |

^a^ Disease classification corresponding to each code is listed in Supplementary Table 1.

^b^ The score was not calculable when both working and absentee hours were 0 in the past 7 days, i.e., the denominator for calculating absenteeism and total work productivity impairment scores was zero.

^c^ A proportion of respondents with the disease and WPAI score calculable.

# **Supplementary Table 5. Percentage of participants with a WPAI-GH score of >0% in terms of absenteeism (A), presenteeism (B), total work productivity impairment (C), and total activity impairment (D) by the period prevalence of diseases (definition 2: diseases and conditions of interests) among female workers**

A) Absenteeism

| **Disease^a^** | **N with disease and score calculable^b^ (A)** | **Period prevalence of disease^c^ (%)** | **N with score not calculable^b^ (B)** | **N with score >0% (C)** | **% with score >0% (C/A+B)** |
| --- | --- | --- | --- | --- | --- |
| Reference | 498 | – | 7 | 29 | 5.7 |
| Back pain | 868 | 10.0 | 16 | 82 | 9.3 |
| Migraine | 262 | 3.0 | 4 | 40 | 15.0 |
| Hearing loss | 263 | 3.0 | 8 | 27 | 10.0 |
| Iron-deficiency anemia | 715 | 8.2 | 8 | 67 | 9.3 |
| Depression | 321 | 3.7 | 6 | 47 | 14.4 |
| Diabetes mellitus | 455 | 5.2 | 3 | 43 | 9.4 |
| Anxiety disorders | 324 | 3.7 | 4 | 37 | 11.3 |
| Femoral neck fracture | 1 | 0 | 0 | 0 | 0 |
| COPD | 17 | 0.2 | 1 | 3 | 16.7 |
| Osteoarthritis | 357 | 4.1 | 9 | 37 | 10.1 |
| Acne vulgaris | 556 | 6.4 | 5 | 60 | 10.7 |
| Disorders of refraction and accommodation | 2,897 | 33.2 | 43 | 207 | 7.0 |
| Schizophrenia | 63 | 0.7 | 2 | 10 | 15.4 |
| Bronchial asthma | 658 | 7.6 | 6 | 68 | 10.2 |
| Ischemic stroke | 45 | 0.5 | 1 | 1 | 2.2 |
| Atopic dermatitis | 543 | 6.2 | 7 | 58 | 10.5 |
| Dysthymia | 24 | 0.3 | 1 | 2 | 8.0 |
| Alcohol use disorder | 2 | 0 | 0 | 0 | 0 |
| Bipolar disorder | 51 | 0.6 | 2 | 7 | 13.2 |
| Oral-related diseases | 5,435 | 62.4 | 69 | 419 | 7.6 |
| Epilepsy | 55 | 0.6 | 0 | 5 | 9.1 |
| Diarrheal disorders | 615 | 7.1 | 9 | 84 | 13.5 |
| Tension headache | 85 | 1.0 | 2 | 12 | 13.8 |
| Ischemic heart disease | 5 | 0.1 | 0 | 1 | 20.0 |
| Sensory organ diseases | 4,069 | 46.7 | 50 | 308 | 7.5 |
| Neck stiffness | 129 | 1.5 | 2 | 18 | 13.7 |
| Diseases of esophagus, stomach and duodenum | 2,262 | 26.0 | 28 | 238 | 10.4 |
| Constipation | 836 | 9.6 | 15 | 93 | 10.9 |
| Obesity | 46 | 0.5 | 1 | 7 | 14.9 |
| Hypertension | 727 | 8.3 | 5 | 67 | 9.2 |
| Hyperlipidemia | 1,183 | 13.6 | 14 | 86 | 7.2 |
| Infectious diseases | 1,255 | 14.4 | 18 | 109 | 8.6 |
| Pollinosis | 135 | 1.5 | 4 | 13 | 9.4 |
| Menstrual disorders | 622 | 7.1 | 11 | 61 | 9.6 |
| Menopausal disorders | 475 | 5.5 | 7 | 49 | 10.2 |
| Uterine cancer | 53 | 0.6 | 0 | 4 | 7.5 |
| Breast cancer | 204 | 2.3 | 4 | 26 | 12.5 |
| Prostate cancer | 0 | – | 0 | 0 | – |
| Colon cancer | 31 | 0.4 | 0 | 4 | 12.9 |
| Gastric cancer | 11 | 0.1 | 0 | 3 | 27.3 |
| Liver cancer | 1 | 0 | 0 | 0 | 0 |
| Lung cancer | 5 | 0.1 | 0 | 0 | 0 |
| Insomnia | 558 | 6.4 | 10 | 78 | 13.7 |
| Allergic rhinitis | 2,289 | 26.3 | 25 | 200 | 8.6 |

B) Presenteeism

| **Disease^a^** | **N with disease and score calculable^b^ (A)** | **Period prevalence of disease^c^ (%)** | **N with score not calculable^b^ (B)** | **N with score >0% (C)** | **% with score >0% (C/A+B)** |
| --- | --- | --- | --- | --- | --- |
| Reference | 505 | – | 0 | 199 | 39.4 |
| Back pain | 884 | 10.1 | 0 | 444 | 50.2 |
| Migraine | 266 | 3.1 | 0 | 173 | 65.0 |
| Hearing loss | 271 | 3.1 | 0 | 146 | 53.9 |
| Iron-deficiency anemia | 723 | 8.3 | 0 | 338 | 46.7 |
| Depression | 327 | 3.8 | 0 | 218 | 66.7 |
| Diabetes mellitus | 458 | 5.3 | 0 | 228 | 49.8 |
| Anxiety disorders | 328 | 3.8 | 0 | 192 | 58.5 |
| Femoral neck fracture | 1 | 0 | 0 | 0 | 0 |
| COPD | 18 | 0.2 | 0 | 8 | 44.4 |
| Osteoarthritis | 366 | 4.2 | 0 | 174 | 47.5 |
| Acne vulgaris | 561 | 6.4 | 0 | 293 | 52.2 |
| Disorders of refraction and accommodation | 2,940 | 33.7 | 0 | 1,440 | 49.0 |
| Schizophrenia | 65 | 0.7 | 0 | 43 | 66.2 |
| Bronchial asthma | 664 | 7.6 | 0 | 336 | 50.6 |
| Ischemic stroke | 46 | 0.5 | 0 | 16 | 34.8 |
| Atopic dermatitis | 550 | 6.3 | 0 | 296 | 53.8 |
| Dysthymia | 25 | 0.3 | 0 | 11 | 44.0 |
| Alcohol use disorder | 2 | 0 | 0 | 2 | 100 |
| Bipolar disorder | 53 | 0.6 | 0 | 33 | 62.3 |
| Oral-related diseases | 5,504 | 63.2 | 0 | 2,571 | 46.7 |
| Epilepsy | 55 | 0.6 | 0 | 35 | 63.6 |
| Diarrheal disorders | 624 | 7.2 | 0 | 330 | 52.9 |
| Tension headache | 87 | 1.0 | 0 | 58 | 66.7 |
| Ischemic heart disease | 5 | 0.1 | 0 | 3 | 60.0 |
| Sensory organ diseases | 4,119 | 47.3 | 0 | 2,008 | 48.7 |
| Neck stiffness | 131 | 1.5 | 0 | 69 | 52.7 |
| Diseases of esophagus, stomach and duodenum | 2,290 | 26.3 | 0 | 1,173 | 51.2 |
| Constipation | 851 | 9.8 | 0 | 434 | 51.0 |
| Obesity | 47 | 0.5 | 0 | 20 | 42.6 |
| Hypertension | 732 | 8.4 | 0 | 326 | 44.5 |
| Hyperlipidemia | 1,197 | 13.7 | 0 | 529 | 44.2 |
| Infectious diseases | 1,273 | 14.6 | 0 | 624 | 49.0 |
| Pollinosis | 139 | 1.6 | 0 | 77 | 55.4 |
| Menstrual disorders | 633 | 7.3 | 0 | 359 | 56.7 |
| Menopausal disorders | 482 | 5.5 | 0 | 233 | 48.3 |
| Uterine cancer | 53 | 0.6 | 0 | 28 | 52.8 |
| Breast cancer | 208 | 2.4 | 0 | 80 | 38.5 |
| Prostate cancer | 0 | – | 0 | 0 | – |
| Colon cancer | 31 | 0.4 | 0 | 14 | 45.2 |
| Gastric cancer | 11 | 0.1 | 0 | 5 | 45.5 |
| Liver cancer | 1 | 0 | 0 | 0 | 0 |
| Lung cancer | 5 | 0.1 | 0 | 1 | 20.0 |
| Insomnia | 568 | 6.5 | 0 | 342 | 60.2 |
| Allergic rhinitis | 2,314 | 26.6 | 0 | 1,186 | 51.3 |

C) Total work productivity impairment

| **Disease^a^** | **N with disease and score calculable^b^ (A)** | **Period prevalence of disease^c^ (%)** | **N with score not calculable^b^ (B)** | **N with score >0% (C)** | **% with score >0% (C/A+B)** |
| --- | --- | --- | --- | --- | --- |
| Reference | 498 | – | 7 | 210 | 41.6 |
| Back pain | 868 | 10.0 | 16 | 458 | 51.8 |
| Migraine | 262 | 3.0 | 4 | 176 | 66.2 |
| Hearing loss | 263 | 3.0 | 8 | 150 | 55.4 |
| Iron-deficiency anemia | 715 | 8.2 | 8 | 355 | 49.1 |
| Depression | 321 | 3.7 | 6 | 221 | 67.6 |
| Diabetes mellitus | 455 | 5.2 | 3 | 235 | 51.3 |
| Anxiety disorders | 324 | 3.7 | 4 | 196 | 59.8 |
| Femoral neck fracture | 1 | 0 | 0 | 0 | 0 |
| COPD | 17 | 0.2 | 1 | 8 | 44.4 |
| Osteoarthritis | 357 | 4.1 | 9 | 184 | 50.3 |
| Acne vulgaris | 556 | 6.4 | 5 | 305 | 54.4 |
| Disorders of refraction and accommodation | 2,897 | 33.2 | 43 | 1,479 | 50.3 |
| Schizophrenia | 63 | 0.7 | 2 | 44 | 67.7 |
| Bronchial asthma | 658 | 7.6 | 6 | 353 | 53.2 |
| Ischemic stroke | 45 | 0.5 | 1 | 16 | 34.8 |
| Atopic dermatitis | 543 | 6.2 | 7 | 307 | 55.8 |
| Dysthymia | 24 | 0.3 | 1 | 10 | 40.0 |
| Alcohol use disorder | 2 | 0 | 0 | 2 | 100 |
| Bipolar disorder | 51 | 0.6 | 2 | 32 | 60.4 |
| Oral-related diseases | 5,435 | 62.4 | 69 | 2,673 | 48.6 |
| Epilepsy | 55 | 0.6 | 0 | 36 | 65.5 |
| Diarrheal disorders | 615 | 7.1 | 9 | 347 | 55.6 |
| Tension headache | 85 | 1.0 | 2 | 61 | 70.1 |
| Ischemic heart disease | 5 | 0.1 | 0 | 3 | 60.0 |
| Sensory organ diseases | 4,069 | 46.7 | 50 | 2,070 | 50.3 |
| Neck stiffness | 129 | 1.5 | 2 | 70 | 53.4 |
| Diseases of esophagus, stomach and duodenum | 2,262 | 26.0 | 28 | 1,212 | 52.9 |
| Constipation | 836 | 9.6 | 15 | 447 | 52.5 |
| Obesity | 46 | 0.5 | 1 | 23 | 48.9 |
| Hypertension | 727 | 8.3 | 5 | 346 | 47.3 |
| Hyperlipidemia | 1,183 | 13.6 | 14 | 554 | 46.3 |
| Infectious diseases | 1,255 | 14.4 | 18 | 644 | 50.6 |
| Pollinosis | 135 | 1.5 | 4 | 78 | 56.1 |
| Menstrual disorders | 622 | 7.1 | 11 | 363 | 57.3 |
| Menopausal disorders | 475 | 5.5 | 7 | 243 | 50.4 |
| Uterine cancer | 53 | 0.6 | 0 | 28 | 52.8 |
| Breast cancer | 204 | 2.3 | 4 | 87 | 41.8 |
| Prostate cancer | 0 | – | 0 | 0 | – |
| Colon cancer | 31 | 0.4 | 0 | 16 | 51.6 |
| Gastric cancer | 11 | 0.1 | 0 | 6 | 54.5 |
| Liver cancer | 1 | 0 | 0 | 0 | 0 |
| Lung cancer | 5 | 0.1 | 0 | 1 | 20.0 |
| Insomnia | 558 | 6.4 | 10 | 347 | 61.1 |
| Allergic rhinitis | 2,289 | 26.3 | 25 | 1,230 | 53.2 |

D)Total activity impairment

| **Disease^a^** | **N with disease and score calculable^b^ (A)** | **Period prevalence of disease^c^ (%)** | **N with score not calculable^b^ (B)** | **N with score >0% (C)** | **% with score >0% (C/A+B)** |
| --- | --- | --- | --- | --- | --- |
| Reference | 505 | – | 0 | 207 | 41.0 |
| Back pain | 884 | 10.1 | 0 | 490 | 55.4 |
| Migraine | 266 | 3.1 | 0 | 177 | 66.5 |
| Hearing loss | 271 | 3.1 | 0 | 152 | 56.1 |
| Iron-deficiency anemia | 723 | 8.3 | 0 | 361 | 49.9 |
| Depression | 327 | 3.8 | 0 | 235 | 71.9 |
| Diabetes mellitus | 458 | 5.3 | 0 | 249 | 54.4 |
| Anxiety disorders | 328 | 3.8 | 0 | 210 | 64.0 |
| Femoral neck fracture | 1 | 0 | 0 | 0 | 0 |
| COPD | 18 | 0.2 | 0 | 9 | 50.0 |
| Osteoarthritis | 366 | 4.2 | 0 | 199 | 54.4 |
| Acne vulgaris | 561 | 6.4 | 0 | 291 | 51.9 |
| Disorders of refraction and accommodation | 2,940 | 33.7 | 0 | 1,512 | 51.4 |
| Schizophrenia | 65 | 0.7 | 0 | 47 | 72.3 |
| Bronchial asthma | 664 | 7.6 | 0 | 359 | 54.1 |
| Ischemic stroke | 46 | 0.5 | 0 | 18 | 39.1 |
| Atopic dermatitis | 550 | 6.3 | 0 | 311 | 56.5 |
| Dysthymia | 25 | 0.3 | 0 | 14 | 56.0 |
| Alcohol use disorder | 2 | 0 | 0 | 2 | 100 |
| Bipolar disorder | 53 | 0.6 | 0 | 37 | 69.8 |
| Oral-related diseases | 5,504 | 63.2 | 0 | 2,720 | 49.4 |
| Epilepsy | 55 | 0.6 | 0 | 35 | 63.6 |
| Diarrheal disorders | 624 | 7.2 | 0 | 342 | 54.8 |
| Tension headache | 87 | 1.0 | 0 | 61 | 70.1 |
| Ischemic heart disease | 5 | 0.1 | 0 | 3 | 60.0 |
| Sensory organ diseases | 4,119 | 47.3 | 0 | 2,116 | 51.4 |
| Neck stiffness | 131 | 1.5 | 0 | 72 | 55.0 |
| Diseases of esophagus, stomach and duodenum | 2,290 | 26.3 | 0 | 1,265 | 55.2 |
| Constipation | 851 | 9.8 | 0 | 477 | 56.1 |
| Obesity | 47 | 0.5 | 0 | 24 | 51.1 |
| Hypertension | 732 | 8.4 | 0 | 356 | 48.6 |
| Hyperlipidemia | 1,197 | 13.7 | 0 | 569 | 47.5 |
| Infectious diseases | 1,273 | 14.6 | 0 | 670 | 52.6 |
| Pollinosis | 139 | 1.6 | 0 | 78 | 56.1 |
| Menstrual disorders | 633 | 7.3 | 0 | 373 | 58.9 |
| Menopausal disorders | 482 | 5.5 | 0 | 262 | 54.4 |
| Uterine cancer | 53 | 0.6 | 0 | 25 | 47.2 |
| Breast cancer | 208 | 2.4 | 0 | 93 | 44.7 |
| Prostate cancer | 0 | – | 0 | 0 | – |
| Colon cancer | 31 | 0.4 | 0 | 15 | 48.4 |
| Gastric cancer | 11 | 0.1 | 0 | 6 | 54.5 |
| Liver cancer | 1 | 0 | 0 | 0 | 0 |
| Lung cancer | 5 | 0.1 | 0 | 1 | 20.0 |
| Insomnia | 568 | 6.5 | 0 | 367 | 64.6 |
| Allergic rhinitis | 2,314 | 26.6 | 0 | 1,242 | 53.7 |

^a^ Disease classification corresponding to each code is listed in Supplementary Table 1.

^b^ The score was not calculable when both working and absentee hours were 0 in the past 7 days, i.e., the denominator for calculating absenteeism and total work productivity impairment scores was zero.

^c^ A proportion of respondents with the disease and WPAI score calculable.

# **Supplementary Table 6. Monthly absenteeism cost by participant characteristics**

| **Characteristics** | | **n** | **Monthly absenteeism cost**^a^ | |
| --- | --- | --- | --- | --- |
|  |  |  | **Mean (SD)** | **Median (Q1, Q3)** |
| Overall |  | 31,227 | 5,247.7 (29,109.1) | 0 (0, 0) |
| Sex/ age category | Male | 22,610 | 5,569.3 (31,405.2) | 0 (0, 0) |
|  | Male, ≤ 29 years old | 1,074 | 3,587.3 (18,164.2) | 0 (0, 0) |
|  | Male, 30–49 years old | 9,056 | 5,625.7 (30,944.8) | 0 (0, 0) |
|  | Male, ≥ 50 years old | 12,480 | 5,699.0 (32,609.1) | 0 (0, 0) |
|  | Female | 8,617 | 4,403.8 (21,950.8) | 0 (0, 0) |
|  | Female, ≤ 29 years old | 766 | 3,357.6 (18,616.9) | 0 (0, 0) |
|  | Female, 30–49 years old | 4,484 | 4,719.9 (22,145.2) | 0 (0, 0) |
|  | Female, ≥ 50 years old | 3,367 | 4,220.9 (22,383.7) | 0 (0, 0) |
| Job category | Professional/technical workers | 7,745 | 5,190.0 (27,398.7) | 0 (0, 0) |
|  | Managerial positions | 5,608 | 4,144.3 (27,977.5) | 0 (0, 0) |
|  | Clerical workers | 7,917 | 4,698.3 (26,345.5) | 0 (0, 0) |
|  | Marketing workers | 2,815 | 5,404.5 (31,248.7) | 0 (0, 0) |
|  | Sales workers | 235 | 7,830.6 (39,782.2) | 0 (0, 0) |
|  | Transportation and communication workers | 63 | 7,071.5 (32,365.3) | 0 (0, 0) |
|  | Security workers | 95 | 6,378.4 (34,681.0) | 0 (0, 0) |
|  | Skilled trade | 1,949 | 7,270.4 (34,741.0) | 0 (0, 0) |
|  | Agriculture, forestry, and fishery | 45 | 8,205.3 (25,744.6) | 0 (0, 0) |
|  | Service (qualification required) | 224 | 6,462.9 (29,745.9) | 0 (0, 0) |
|  | Service (qualification not required) | 992 | 5,658.4 (29,940.9) | 0 (0, 0) |
|  | Others | 3,539 | 6,648.9 (33,511.5) | 0 (0, 0) |

Q1, first quartile; Q3, third quartile; SD, standard deviation

^a^ Calculated using the formula: absenteeism score (percentage) ×average monthly salary by sex and age (based on the National Basic Survey on Wage Structure, <https://www.mhlw.go.jp/toukei/itiran/roudou/chingin/kouzou/z2022/dl/13.pdf>, available in Japanese)/12

# **Supplementary Table 7. Monthly presenteeism cost by participant characteristics**

| **Characteristics** | | **n** | **Monthly presenteeism cost^a^** | |
| --- | --- | --- | --- | --- |
|  |  |  | **Mean ± SD** | **Median (Q1, Q3)** |
| Overall |  | 31,540 | 39,678.5 (69,154.7) | 0, (0, 55,120) |
| Sex/ age category | Male | 22,825 | 39,935.8 (72,594.4) | 0, (0, 41,650) |
|  | Male, ≤ 29 years old | 1,083 | 36,942.6 (56,944.9) | 0, (0, 51,860) |
|  | Male, 30–49 years old | 9,138 | 46,563.4 (76,401.4) | 0, (0, 72,720) |
|  | Male, ≥ 50 years old | 12,604 | 35,387.9 (70,571.1) | 0, (0, 41,650) |
|  | Female | 8,715 | 39,004.5 (59,205.1) | 0, (0, 55,700) |
|  | Female, ≤ 29 years old | 768 | 46,589.4 (57,886.3) | 24,080 (0, 72,240) |
|  | Female, 30–49 years old | 4,543 | 44,052.6 (63,478.6) | 0, (0, 55,700) |
|  | Female, ≥ 50 years old | 3,404 | 30,555.9 (52,191.4) | 0, (0, 47,460) |
| Job category | Professional/technical workers | 7,816 | 39,341.9 (68,824.1) | 0, (0, 53,640) |
|  | Managerial positions | 5,657 | 34,333.9 (68,138.3) | 0, (0, 41,090) |
|  | Clerical workers | 7,988 | 41,175.3 (66,125.3) | 0, (0, 55,840) |
|  | Marketing workers | 2,838 | 47,178.5 (76,451.8) | 0, (0, 67,160) |
|  | Sales workers | 239 | 37,517.4 (69,685.4) | 0, (0, 41,650) |
|  | Transportation and communication workers | 66 | 33,631.5 (55,522.8) | 0, (0, 41,090) |
|  | Security workers | 96 | 41,228.3 (78,397.4) | 0, (0, 69,940) |
|  | Skilled trade | 1,975 | 42,213.3 (73,478.2) | 0, (0, 59,400) |
|  | Agriculture, forestry, and fishery | 45 | 69,873.1 (81,273.3) | 32,180 (0, 124,950) |
|  | Service (qualification required) | 228 | 34,014.4 (58,073.7) | 0, (0, 52,220) |
|  | Service (qualification not required) | 1,011 | 38,210.1 (68,667.1) | 0, (0, 41,650) |
|  | Others | 3,581 | 38,785.1 (69,358.7) | 0, (0, 49,180) |

Q1, first quartile; Q3, third quartile; SD, standard deviation;

^a^ Calculated using the formula: presenteeism score (percentage) ×the average annual salary by sex and age (based on the National Basic Survey on Wage Structure <https://www.mhlw.go.jp/toukei/itiran/roudou/chingin/kouzou/z2022/dl/13.pdf>, available in Japanese)/12
